# New pharmacological agents and novel cardiovascular pharmacotherapy strategies in 2024

**DOI:** 10.1093/ehjcvp/pvaf012

**Published:** 2025-03-08

**Authors:** Juan Tamargo, Stefan Agewall, Giuseppe Ambrosio, Claudio Borghi, Elisabetta Cerbai, Gheorghe A Dan, Heinz Drexel, Péter Ferdinandy, Erik Lerkevang Grove, Roland Klingenberg, Joao Morais, William Parker, Bianca Rocca, Patrick Sulzgruber, Anne Grete Semb, Samuel Sossalla, Juan Carlos Kaski, Dobromir Dobrev

**Affiliations:** Department of Pharmacology and Toxicology, School of Medicine, Universidad Complutense, Instituto de Investigación Sanitaria Gregorio Marañón, Avenida Ramón y Cajal s/n, 28040 Madrid, Spain; Institute of Clinical Science, Oslo University, 0318 Oslo, Norway; Department of Clinical Sciences, Danderyd Hospital, Karolinska Institute, 182 88 Stockholm, Sweden; Department of Medicine and CERICLET, University of Perugia School of Medicine, 06156 Perugia, Italy; Department of Cardiovascular Medicine, University of Bologna-IRCCS AOU S. Orsola, 40138 Bologna, Italy; Department Neurofarba, Section of Pharmacology and Toxicology, University of Florence, 50121 Firenze, Italy; Carol Davila. University of Medicine, Academy of Romanian Scientist, Bucharest, Sector 2, Romania; Vorarlberg Institute for Vascular Investigation & Treatment (VIVIT), 6800 Feldkirch, Austria; Department of Pharmacology and Pharmacotherapy, Semmelweis University, Budapest, H-1089, Hungary; Pharmahungary Group, Szeged H-6722, Hungary; Center for Pharmacology and Drug Research & Development, Semmelweis University, Budapest, H-1089, Hungary; Department of Cardiology, Aarhus University Hospital, Aarhus 8200, Denmark; Department of Clinical Medicine, Faculty of Health, Aarhus University, Aarhus 8200, Denmark; Department of Cardiology, Kerckhoff Heart and Thorax Center, 61231 Bad Nauheim, Germany; ciTechCare—Center for Innovative Care and Health Technology, Polytechnic University of Leiria, 2414-016 Leira, Portugal; Cardiovascular Research Unit, University of Sheffield, Sheffield S5 7AU, UK; Department of Medicine and Surgery, LUM University, 70010 Casamassima, Bari, Italy; Department of Medicine, Division of Cardiology, Medical University of Vienna, 1090 Vienna, Austria; Preventive Cardio-Rheuma clinic, Division of Research and Innovation, REMEDY centre, Diakonhjemmet Hospital, 0370 Oslo, Norway; Medical Clinic I, Cardiology and Angiology, Justus-Liebig-University, Giessen, Germany; Department of Cardiology, Kerckhoff-Clinic/DZHK, 61231 Bad Nauheim, Germany; Molecular and Clinical Sciences Research Institute, St. George's, University of London, Cranmer Terrace, London SW17 0RE, UK; Institute of Pharmacology, West-German Heart and Vascular Centre, University Duisburg-Essen, 45122 Essen, Germany; Department of Medicine, Montreal Heart Institute and Université de Montréal, Montréal, Quebec H1T 1C8, Canada; Department of Integrative Physiology, Baylor College of Medicine, Houston, TX 77030, USA

**Keywords:** Cardiovascular drugs, New cardiovascular drugs, Drug combinations, Drug interactions and safety, Cardiovascular pharmacotherapy, Cardiovascular pharmacological strategies

## Abstract

Despite substantial advances in cardiovascular pharmacotherapy and devices in recent years, prevention and treatment of many cardiovascular diseases (CVDs) remain limited, thus reflecting the need for more effective and safer pharmacological strategies. In this review, we summarize the most relevant studies in cardiovascular pharmacotherapy in 2024, including the approval of first-in-class drugs for the treatment of resistant hypertension and pulmonary arterial hypertension, label expansions for bempedoic acid and semaglutide, and the results of major randomized clinical trials (RCTs) that have met the pre-specified primary endpoints, thereby filling some gaps in knowledge and opening new perspectives in the management of CVD, and those RCTs whose results did not confirm the proposed research hypotheses. We also include a section on drug safety, where we describe the newest data on adverse reactions and drug–drug interactions that may complicate treatment and/or reduce drug adherence with the consequent decrease in drug effectiveness. Finally, we present the most important ongoing phase 2 and phase 3 clinical trials assessing the efficacy and safety of cardiovascular drugs for the prevention and treatment of CVD.

## Graphical Abstract

**Graphical Abstract:**
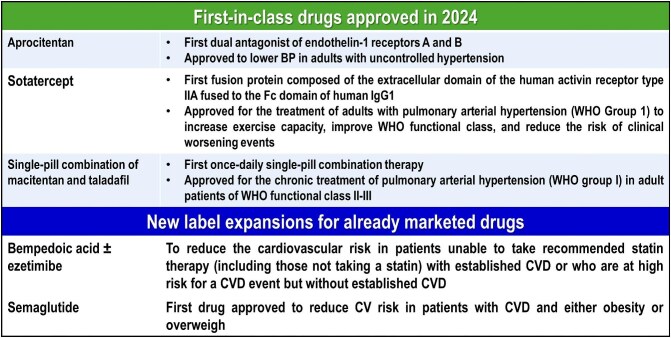

## Introduction

Cardiovascular diseases (CVDs) remain the leading cause of death and disability worldwide, and despite important advances, treatment of many CVDs remains suboptimal. Sometimes the safety profile of cardiovascular (CV) drugs, namely adverse effects and drug–drug interactions, precludes their clinical use in many patients or leads to poor adherence that may contribute to poorer clinical outcomes and safety. Hence, there is a need for newer, more efficacious and safer agents for the treatment of CVD. In 2024, three ‘first-in-class’ drugs were approved: aprocitentan, an endothelin-1 (ET-1) receptor antagonist (ERA) for the treatment of uncontrolled arterial hypertension, and the fusion protein sotatercept, and the fixed-dose combination of macitentan and tadalafil (M/T-FDC) for the treatment of pulmonary arterial hypertension (PAH). Two already approved drugs, semaglutide and bempedoic acid, received important label expansions, and several randomized clinical trials (RCTs) evaluated the efficacy and safety of drugs already marketed to open new pharmacological options and fill unmet needs for the management of CVD.

For the selection of the most relevant advances in CV pharmacology in 2024, we reviewed individual drugs or fixed combinations, new indications or approval of drugs in the websites from the US Food and Drug Administration (FDA) and the European Medicines Agency; searched for clinical trials in pre-specified fields published in 2024 using the MEDLINE/PubMed and Embase databases and analysed the advances in CV pharmacotherapy presented at the Annual Scientific Sessions of the European Society of Cardiology, American College of Cardiology, and American Heart Association. Only high-quality clinical trials were considered.

To simplify the presentation of large amounts of data, clinical trials were grouped as those that achieved the primary endpoint(s) (‘positive trials’), those which did not prove their primary endpoint(s) (‘negative or neutral trials’), and studies that analysed the safety profiles (adverse reactions and drug–drug interactions) of several drugs administered in patients with CVD. The main characteristics and findings of the selected trials are summarized in *Tables 1*–*3*.

**Table 1:** Clinical trials with positive results^a^

Trial^b^	Trial design/population	Treatment	Primary endpoint	Results
ADALA^23^ NCT05632445	P, OL, R trial. 90 after successful LAAO (59% with previous major bleeding events) Female: FU: 3 months	Apixaban (2.5 and 5 mg b.i.d.) vs. DAPT (100 mg aspirin plus clopidogrel 75 mg o.d.) for 3 months	Thrombo-embolic events, including stroke, systemic embolism, and device-related thrombosis (DRT) and major bleeding	Low-dose DOAC reduced the primary endpoint (HR 0.19; *P* = 0.02) and the rate of DRT (0% vs. 8.7%; *P* = 0.04), but there were no differences in major bleeding or thrombo-embolic events between groups
A DUE trial^6^ NCT03904693	Phase 3, R, DB, PD, adaptive study. 187 with PAH (WHO functional class II and III) Female: 78% FU: 16 weeks	FDC of macitentan and tadalafil (M/T-FDC) vs. macitentan 10 mg and vs. tadalafil 40 mg monotherapies	Change in pulmonary vascular resistance (PVR) at week 16	The PVR reduction with M/T-FDC was significantly greater than with macitentan or tadalafil monotherapy (both *P* < 0.0001) Anaemia, hypotension and oedema were more frequent with M/T-FDC
ANNEXA-I^27^ NCT03661528	Phase 4, unblinded, R trial. 530 taking a factor Xa inhibitor within 15 h before having an acute intracerebral haemorrhage Female: 45% FU: 12 h	Andexanet alfa (a high-dose i.v. bolus or a low-dose bolus over 15-30 min followed by an i.v. infusion over 2 h) vs. usual care	Haemostatic efficacy: expansion of haematoma volume by ≤35% and an increase in the score on the NIHSS of <7 points at 12 h, and no receipt of rescue therapy between 3 and 12 h	Haemostatic efficacy was more often achieved in the andexanet group (*P* = 0.003) resulting in better control of haematoma expansion. However, thrombotic events, including ischaemic stroke increased in the andexanet group (*P* = 0.048). There were no differences between the groups in the score on the modified Rankin scale or in death within 30 days
ARTESIA^16^ NCT01938248	Phase 4, R, DB, DD trial. 4012 with device-detected subclinical AF Female: 36% FU: 3.5 years	Apixaban (5 mg b.i.d.; 2.5 mg b.i.d. if ≥2 of: age >80, weight ≤60 kg, or sCr ≥133 mmol/L) vs. aspirin (81 mg o.d.)	Composite of stroke and systemic embolism, was assessed in the intention-to-treat population	As compared with aspirin, apixaban reduced the risk of stroke or systemic embolism, ischaemic stroke, and stroke from any cause but increased the rate of major bleeding (1.80; 1.26–2.57; *P* = 0.001)
ATTRibute-CM Investigator^45^ NCT03860935	Phase 3, DB, R trial. 632 with ATTR-CM, clinical HF and eGFR ≥30 mL/min/1.73 m^2^ Female: 10% FU: 30 months	Acoramidis (800 mg b.i.d.) vs. placebo	Hierarchical analysis of all-cause mortality, CVH, change from baseline in the NT-proBNP level, and 6MWD	Acoramidis improved the primary outcome over placebo (win ratio 1.8; 1.4–2.2; *P* < 0.0001). The associated win ratios for all-cause mortality and CV-related hospitalization and for all-cause mortality from any cause, CV-related hospitalization and 6MWD favoured acoramidis
BPROAD^54^ NCT03808311	R, PD trial. 12 821 adults with increased SBP and increased risk of CVD Female: 45% FU: 5 years	Intensive treatment targeted an SBP <120 mm Hg or standard treatment targeted to an SBP <140 mm Hg	Composite of non-fatal stroke, non-fatal MI, treatment or hospitalization for HF, or death from CV causes	The incidence of major CV death events was lower in the intensive treatment than in the standard treatment group (HR 0.79; 0.69–0.90; *P* < 0.001). Symptomatic hypotension and hyperkalaemia occurred more frequently in the intensive treatment group
CLEAR Outcomes^11^ NCT02993406	Phase 3, R, DB, PC trial. 13 970, baseline LDL-C ≥2.59 mmol/L and DM, pre-DM or normoglycaemia Female: 49% FU: 3.4 years	Bempedoic acid 180 mg o.d. or placebo	Time to first occurrence of MACE-4 events, stratified by basal glycaemia status and primary or secondary prevention status	In diabetic patients, bempedoic acid reduced LDL-C (21%) and MACE-4 (17%), with similar trends among patients with pre-DM or normoglycaemia. In patients without DM, bempedoic acid did not increase HbA1c, glucose levels, or the incidence of new-onset diabetes
COLCOT^43^ NCT02551094	Pre-specified subgroup analysis of the trial. 959 with T2D and recent MI Female: 22% FU: 22.6 months	Colchicine (0.5 mg/day) vs. PCB initiated within 30 days after an MI	Composite of CV death, resuscitated cardiac arrest, MI, stroke, or urgent hospitalization for angina requiring coronary revascularization	The risk of the primary endpoint event occurred less frequently in the colchicine than in the placebo group (HR 0.65; 0.44–0.96; *P* = 0.03). Significantly more nausea and pneumonia with colchicine
COLOCT^42^ NCT04848857	P, R, DB, single-centre trial. 128 with ACS and lipid-rich plaque (lipid pool arc >90°) Female: 33% FU: 1 year	Colchicine (0.5 mg o.d.) or placebo	Change in minimal fibrous cap thickness from baseline to the 12-month follow-up	Compared with placebo, colchicine increased the minimal fibrous cap thickness (*P* = 0.006) and decreased reduced average lipid arc, mean angular extension of macrophages, high-sensitivity C-reactive protein interleukin-6, and myeloperoxidase level
DACAB-FE^25^ NCT03987373	P, OL, R, controlled trial. 500 with elective CABG who completed the DACAB trial Female: 18.2% FU: 5 years	Ticagrelor 90 mg b.i.d. plus aspirin 100 mg o.d., ticagrelor alone or aspirin alone	MACE (composite of all-cause mortality, MI, stroke, and coronary revascularization)	Risk of MACE at 5 years after CABG was significantly lower with DAPT vs. aspirin monotherapy (HR 0.65, 0.43–0.99; *P* = 0.04) or ticagrelor monotherapy (0.66, 0.44–1.00; *P* = 0.05)
EPIC-CAD^18^ NCT03718559	Phase 4, OL, adjudicator-masked, R trial. 524 with AF and stable CAD Mean CHA2DS2-VASc score 4.3 Female: 24% FU: 12 months	Edoxaban (60 mg o.d.) vs. edoxaban plus aspirin or a P2Y12 inhibitor, at the prescriber discretion	Composite of all-cause mortality, MI, stroke, systemic embolism, unplanned urgent revascularization, and major or clinically relevant non-major bleeding	Edoxaban monotherapy led to a lower risk of the primary endpoint (HR 0.44; 0.30–0.65; *P* < 0.001) and major bleeding or clinically relevant non-major bleeding (0.34; 0.22–0.53). The cumulative incidence of major ischaemic events was similar in both groups
ESPRIT^55^ NCT04030234	OL, R, controlled trial. 11 255 (4359 with DM, 3022 with previous stroke) Female: 41.3% FU: 3.4 years	Intensive treatment targeting standard office SBP <120 mm Hg or standard treatment targeting <140 mm Hg	Composite of MI, revascularization, HF hospitalization, stroke, or CV death, assessed by the intention-to-treat principle	Targeting SBP of <120 mm Hg, as compared with that of <140 mm Hg prevents major vascular events (HR 0.88; CI 0.78–0.99; *P* = 0.028), with minor excess risk
FAST-MI program^12^ NCT00673036, NCT01237418, NCT02566200	Nationwide French surveys. 2258 aged ≥80 years admitted for AMI ≤48 h from onset Female: 51% FU: 5 years	Discharged without LLT (18%), with conventional doses (38%) and with high-dose LLT (43%)	5-year mortality in patients aged ≥80 years discharged alive	High-intensity, but not conventional-intensity LLT at discharge after AMI, reduced all-cause mortality at 5 years in older adults, suggesting that high-intensity LLT should not be denied on the basis of old age
FINEARTS-HF^49^ NCT04435626	Phase 3, DB, R trial. 6016 patients with HFmrEF or HFpEF Female: 45.5% FU: 32 months	Finerenone (maximal dose 20 or 40 mg depending on the baseline eGFR)	Composite of worsening HF events (first/recurrent unplanned hospitalization or urgent visit for HF) and CV death	Finerenone reduced the rate of the primary composite endpoint (RR 0.84; 0.74–0.95; *P* = 0.007) or the total number of worsening HF events (0.82; 0.71–0.94; *P* = 0.006) but not of CV death vs. placebo
FLOW^31^ NCT03819153	Phase 3, R, DB, PD trial. 3533 patients with T2D and CKD Female: 30% FU: 3.4 years	s.c. semaglutide (1.0 mg) or placebo once weekly, plus RAAS blockade	Onset of kidney failure (dialysis, transplantation, or eGFR of <15 mL/min/1.73 m^2^), ≥50% reduction in eGFR from baseline, or renal-related or CV death	Semaglutide reduced the risk of major kidney disease events vs. placebo (HR 0.76; 0.66–0.88; *P* = 0.0003), kidney-specific components of the primary outcome (0.79; 0.66–0.94), and CV death (0.71; 0.56–0.89). The trial was stopped early for efficacy
HELIOS-B^47^ Fontana *et al*. NEJM NCT04153149	Phase 3, R, DB, PC trial. 655 with ATTR-CM (either variant or wild-type) Female: 8% FU: 48 months	Vutrisiran (25 mg s.c. once every 3 months) vs. placebo for up to 36 months 40% of patients received tafamidis at baseline	Composite of all-cause mortality and recurrent CV events	Compared with placebo, vutrisiran reduced the risk of all-cause mortality and recurrent CV events (*P* = 0.02) and all-cause mortality throughout 42 months in the overall population (0.64; 0.46–0.90; *P* = 0.01). Vutrisiran also improved the 6MWD and the KCCQ-OS score vs. placebo
MICON^14^	16 373 patients with IS/TIA; 4668 had ≥1 CMBs Female: 42.5% FU: 1.34 years	Users vs. non-users of statin therapy	Occurrence of recurrent symptomatic IS or intracranial haemorrhage (ICrH)	Compared with non-users, statins reduced the risk of any stroke (aHR 0.68; 0.56–0.84) and IS (0.65; 0.51–0.82), without an increased risk of IcrH. Results were consistent when considering anatomical distribution and high burden (≥5) of CMBs
OPT-RISK^22^ NCT03431142	Phase 4, DB, R, PC trial. 7758 with ACS undergoing PCI at high bleeding and ischaemic risk who completed 9–12 months of DAPT Female: 41% FU: 9 months	Clopidogrel or DAPT (aspirin + clopidogrel) for 9 months following completion of 9–12 months of DAPT after initial PCI	Bleeding (BARC types 2, 3, or 5) 9 months after randomization	An extended 9-month clopidogrel monotherapy was superior to DAPT in reducing clinically relevant bleeding (HR 0.75; 0.57–0.97; *P* = 0.03) and MACE (0.74; 0.57–0.96; *P* < 0.001 for non-inferiority; *P* = 0.02 for superiority), without increasing ischaemic events
Rodriguez *et al*.^39^	Propensity-matched, cohort study. 41 222 with overweight or obesity identified using EHR data 52.0% had T2D Female: 70.5%	Semaglutide (0.5 mg o.d.) vs. tirzepatide (5 mg o.d.)	Weight loss assessed as hazard of achieving ≥5%, ≥10%, and ≥15% weight loss, and per cent change in weight at 3, 6, and 12 months	In individuals with or without T2D, use of tirzepatide produced significantly greater weight loss and larger reductions in BW than semaglutide
Subanalysis of the SELECT trial^29^ NCT03574597	Phase 3, R, DB, PC, event-driven trial. 4286 with a BMI ≥27 kg/m², ASCVD, and HF (HFrEF, HFpEF, or unclassified HF) Female: 27% FU: 39.8 months	Escalating doses of once-weekly s.c. semaglutide over 16 weeks to a target dose of 2.4 mg, or placebo	Composite of HF outcome (CV death or hospitalization or urgent hospital visit for HF); CV death; and all-cause mortality	Semaglutide reduced MACE and composite HF endpoints compared with placebo in those with and without clinical HF, regardless of heart failure subtype and without increased serious adverse events
SUMMIT^53^ NCT04847557	Phase 3, R, DB, PC trial. 731 with HF (LVEF ≥50%) and BMI ≥30 Female: 53% FU: 104 weeks	Tirzepatide (up to 15 mg s.c. once per week) or placebo	Composite of adjudicated CV death or a worsening HF event and the change from baseline to 52 weeks on the KCCQ-CSS	Tirzepatide led to a lower risk of CV death or worsening HF than placebo (HR 0.62; 0.41–0.95; *P* = 0.026), worsening HF events and adjudicated CV death, improved the health status (KCCQ-CSS and 6MWD), and decreased both BW and high-sensitivity C-reactive protein levels
T-PASS^26^ NCT03797651	R, OL, non-inferiority trial. 2850 with ACS (40% with STEMI who underwent drug-eluting stent implantation) Female: 16.5% FU: 12 months	Ticagrelor (90 mg b.i.d.) after <1 month of DAPT (aspirin 100 mg daily + ticagrelor) or 12 months of DAPT	Net clinical benefit, a composite of all-cause death, MI, stent thrombosis, stroke, and major bleeding	Stopping aspirin within 1 month of DAPT followed by ticagrelor monotherapy was non-inferior and provided evidence for superiority to 12 months of ticagrelor-based DAPT for a 1-year primary composite outcome
ULTIMATE-DAPT^21^ NCT03971500	R, DB, PC, PA trial. 3400 with an ACS who completed the IVUS-ACS trial 70% had single-vessel disease FU: 22.6 months Female: 26%	Patients received DAPT (ticagrelor 90 mg b.i.d. + aspirin 100 mg o.d.) for 1 month; then they were randomized to DAPT or ticagrelor + placebo for 11 months	Superiority: bleeding (BARC types 2, 3, or 5) Non-inferiority: MACCE (cardiac death, MI, ischaemic stroke, definite stent thrombosis, or clinically driven target vessel revascularization)	Between 1 and 12 months after PCI, relevant bleeding decreases in the ticagrelor group vs. DAPT (2.1% vs. 4.6%; HR 0.45; 0.30–0.66; *P* < 0.0001) without increasing MACCE. Net adverse clinical events (MACCE or BARC types 1–5 bleeding) were lower in the ticagrelor monotherapy group vs. DAPT group (5.7% vs. 8.2%; *P* = 0.007)
VICTORION-INITIATE^13^ NCT04929249	Phase 3b, P, R, OL, PD trial. 450 with ASCVD, LDL-C ≥70 mg/dL or non-HDL-C ≥100 mg/dL and fasting <500 mg/dL despite statin therapy Female: 31% FU: 330 days	Inclisiran (300 mg s.c. at days 0, 90, and 270) plus usual care vs. usual care alone prescribed at the treating provider's judgement	Percentage change in LDL-C from baseline to day 330 and discontinuation of statin therapy, defined as no statin use ≥30 days before the end-of-study visit	Compared with usual care, the inclisiran first strategy resulted in earlier and greater reductions in LDL-C and more patients achieved LDL-C levels <70 and <55 mg/dL (81.8% vs. 22.2% and 71.6% vs. 8.9%; both *P* < 0.001). Infection-site adverse reactions causing treatment withdrawal occurred more commonly with ‘inclisiran first’ than with usual care

ACS, acute coronary syndrome; AF, atrial fibrillation; aHR, adjusted hazard ratio; AKI, acute kidney injury; AMI, acute myocardial infarction; ASCVD, atherosclerotic cardiovascular disease; ATTR-CM, transthyretin amyloid cardiomyopathy; BARC, Bleeding Academic Research Consortium; b.i.d., twice daily; BMI, body mass index; BW, body weight; CABG, coronary artery bypass grafting; CKD, chronic kidney disease; CMBs, cerebral microbleeds; CV, cardiovascular; CVD, cardiovascular disease; CVH, cardiovascular-related hospitalization; DAPT, dual antiplatelet therapy; DB, double-blind; DD, double-dummy; DKA, diabetic ketoacidosis; DM, diabetes mellitus; eGFR, estimated glomerular filtration rate; EHR, electronic health record; FU, follow-up; HbA1c, glycated haemoglobin; HF, heart failure; HFmeEF/HFpEF/HFrEF, heart failure with mildly reduced/preserved/reduced ejection fraction; HR, hazard ratio; IS, ischaemic stroke; i.v., intravenous; KCCQ-CSS/OS, Kansas City Cardiomyopathy Questionnaire Clinical Summary Score/Overall Summary score; LAAO, left atrial appendage occlusion; LDL-C, LDL cholesterol; LLT: lipid-lowering therapy; LVEF, left ventricular ejection fraction; MACCE, major cardiovascular and cerebrovascular events; MACE, major adverse cardiovascular events; MI, myocardial infarction; MT-FDC, macitentan/sildenafil fixed-dose combination; 6MWD, 6-min walk distance; NCT, ClinicalTrials.gov identifier; NIHSS, National Institutes of Health Stroke Scale; NT-proBNP, N-terminal pro-B-type natriuretic peptide; OL, open label; o.d., once daily; P, prospective; PAH, pulmonary arterial hypertension; PC, placebo-controlled; PCI, percutaneous coronary intervention; PD, parallel-design; PVR, pulmonary vascular resistance; R, randomized; RAAS, renin–angiotensin–aldosterone system; RR, relative risk; SBP, systolic blood pressure; s.c., subcutaneous; sCr, serum creatinine; SGLT2I, sodium–glucose cotransporter-2 inhibitors; STEMI, ST-elevation MI; TIA, transient ischaemic attack; T2D, type 2 diabetes; WHO, World Health Organization.

a In alphabetic order.

b Acronyms of the trials are summarized in Supplementary material online, *Table S1.*

**Table 2:** Clinical trials that did not reach the primary endpoint (negative trials) or provided neutral effects^a^

Trial^b^	Trial design/population	Treatment	Primary endpoint	Results
*Clinical trials with negative results*				
ARCADIA^60^ NCT03192215	Phase 3, R, DB trial. 1015 with cryptogenic stroke and atrial cardiopathy Female: 54.3% FU: 1.8 years	Apixaban, 2.5 or 5 mg b.i.d. vs. aspirin, 81 mg o.d.	Time-to-event analysis was recurrent stroke	The rate of recurrent stroke was similar with apixaban and placebo (4.4% and 4.4%). The trial was stopped for futility after a planned interim analysis
BICS^58^ ISRCTN10497306	R, DB, PC trial. 515 with chronic obstructive pulmonary disease at high risk of exacerbation Female: 47% FU: 12 months	Bisoprolol (1.25 mg o.d. up-titrated to 5 mg/day, using a standardized protocol) vs. placebo	Patient-reported chronic obstructive pulmonary disease exacerbations treated with oral corticosteroids and/or antibiotics during FU	Bisoprolol did not reduce the number of self-reported chronic obstructive pulmonary disease exacerbations requiring treatment with oral corticosteroids and/or antibiotics
CHANCE-3^68^ NCT05439356	Phase 3, DB, R, PC trial. 8343 with a minor-to-moderate IS or TIA and high-sensitivity C-reactive protein ≥2 mg/L Female: 37% FU: 90 days	Colchicine (0.5 mg b.i.d. on days 1–3, followed by 0.5 mg daily thereafter) or placebo	Any new stroke (ischaemic or haemorrhagic) within 90 days after randomization	Low-dose colchicine initiated within 24 h of symptom did not reduce the risk of subsequent stroke (HR 0.98; 0.83–1.16; *P* = 0.79) or increase the risk of serious adverse events as compared with placebo within 90 days
CLEAR SYNERGY^70^ NCT03048825	Phase 3, R, DD, 2-by-2 factorial design trial. 7062 with STEMI (95.2%) or NSTEMI (4.8%) who underwent PCI Female: 20% FU: 3 years	Colchicine (0.5 mg o.d. if <70 kg; 0.5 mg b.i.d. if ≥70 kg; after 90 days, colchicine was given o.d.) vs. placebo	Time-to-event of the composite of CV death, recurrent MI, stroke, or unplanned ischaemia-driven coronary revascularization	Colchicine started soon after MI did not reduce the primary outcome (HR 0.99; 0.85–1.16; *P* = 0.93). The incidence of individual components of the primary outcome was similar in the two groups. Diarrhoea was more frequent with colchicine
CLEAR SYNERGY^73^ NCT03048825		Spironolactone (25 mg o.d.) vs. placebo	Composite 1: CV death or new or worsening HF, evaluated as the total number of events Composite 2: first MI, stroke, new or worsening HF, or CV death	Spironolactone did not reduce the first (aHR 0.91; 0.69–1.21; *P* = 0.51) or the second co-primary outcome (0.96; 0.81–1.13; *P* = 0.60) as compared with placebo. Hyperkalaemia and gynaecomastia were more frequent in the spironolactone group
DAPA-MI^64^ NCT04564742	Phase 3, PG, DB, PC, registry-based trial. 4017 without prior diabetes or chronic HF, presenting with AMI (STEMI/NSTEMI) and impaired LV systolic function Female: 20% FU: 1 year	Dapagliflozin (10 mg o.d.) or placebo	Hierarchical composite of death, HF hospitalization, non-fatal MI, atrial fibrillation/flutter, T2D, NYHA functional class, and BW decrease ≥5% at the last visit	Dapagliflozin had no impact on the composite of CV death or HF hospitalization, but the analysis of the primary hierarchical composite outcome resulted in significantly more wins for dapagliflozin vs. placebo (win ratio, 1.34; 1.20–1.50; *P* < 0.001), mainly driven by the added cardiometabolic outcomes
DICTATE-AHF^74^ NCT04298229	Phase 3, OL, P, R trial. 240 with or without T2D randomized within 24 h of hospitalization for hypervolemic AHF FU: day 5 or discharge	Dapagliflozin 10 mg o.d. vs. usual care with protocolized diuretic titration	Diuretic efficiency expressed as cumulative weight change per cumulative loop diuretic dose, compared across treatment assignment	There were no differences in diuretic efficiency between dapagliflozin and usual care, but dapagliflozin reduced loop diuretic doses (*P* = 0.006) and i.v. diuretic up-titrations (*P ≤* 0.05) to achieve equivalent weight loss as usual care and significantly improved median 24 h natriuresis and urine output
DEPOSITION^62^ NCT03954314	Phase 3, R, DD, blinded trial. 3242 undergoing open cardiac surgery FU: hospital discharge or 10 days Female: 22.3%	IV TXA (5 g) through surgery vs. topical intraoperative TXA (10 g) at the end of surgery	In-hospital seizure	Topical TXA did not reduce seizure incidence, but increased RBC transfusion use and severe haemorrhage requiring ≥4 RBC units. The DSMB stopped the trial for safety concerns
EMPACT-MI^66^ NCT04509674	Phase 3, event-driven, R, DB, PC trial 6522 hospitalized for AMI and at risk for HF Female: 25% FU: 17.9 months	Empagliflozin 10 mg o.d. vs. placebo in addition to standard care within 14 days of admission	Composite of HF hospitalization or all-cause mortality	Empagliflozin did not reduce the primary endpoint vs. placebo (*P* = 0.21), but reduced first (HR, 0.77; 0.60–0.98; *P* = 0.031) and total HF hospitalizations (0.67; 0.51–0.89; *P* = 0.006) across the spectrum of LVEF and congestion risk profiles. Serious AEs were similar in both groups
OCEANIC-AF^61^ NCT05643573	Phase 3, DB, DD, R, PG trial. 14 810 high-risk patients with AF Mean CHA2DS2-VASc score 4.3 Female: 35% FU: 1 year	Asundexian (50 mg o.d.) vs. standard-dose apixaban	Whether asundexian is at least non-inferior to apixaban for the prevention of stroke or systemic embolism	The trial was stopped prematurely because asundexian increased the risk of stroke or systemic embolism vs. apixaban (HR 3.79), but produced fewer major bleeding events (0.32; 0.18–0.55)
REDUCE-AMI^56^ NCT03278509	Phase 4, OL, R, PG, trial. 5020 within 1–7 days post-MI and LVEF ≥50% obtained from the SWEDEHEART and Swedish Population Registries Female: 23% FU: 3.5 years	Metoprolol (median ≥100 mg) or bisoprolol (median ≥5 mg) or no β-blocker (usual care)	Composite of all-cause mortality or MI	Among patients with AMI with HFpEF, long-term β-blocker treatment did not lower the risk of all-cause mortality or MI vs. usual care (7.9% vs.8.3%; HR 0.96; 0.79–1.16; *P* = 0.64) and the cumulative incidence of any of the secondary endpoints
RESPECT-EPA^75^ UMIN000012069	P, R, OL, blinded endpoint trial. 3884 patients with stable CAD and a low EPA/AA ratio (median 0.243) treated with statins for at least 1 month Females: 17% FU: 5 years	EPA (1800 of icosapent ethyl daily) or control group	Composite of CV death, non-fatal MI, non-fatal ischaemic stroke, unstable angina pectoris, and coronary revascularization	The primary endpoint occurred in fewer patients in the EPA group (HR 0.79; 0.62–1.00; *P* = 0.055). The secondary composite endpoint of coronary events was significantly lower in the EPA group (0.73; 0.55–0.97; *P* = 0.03). The rate of new-onset AF was higher in the EPA group (3.1% vs. 1.6%; *P* = 0.017)
*Clinical trials with neutral results*				
AβYSS^57^ NCT03498066	Phase 4, OL, R, non-inferiority trial. 3698 with an MI, LVEF ≥40%, and no history of a CV event in the previous 6 months Female: 17% FU: 3 years	Interruption or continuation of β-blocker treatment	Composite of death, non-fatal MI, non-fatal stroke, or CV hospitalization at the longest follow-up (minimum, 1 year)	Interruption of long-term β-blocker treatment was not found to be non-inferior to a strategy of β-blocker continuation (HR 1.16; *P* = 0.44 for non-inferiority). β-Blocker interruption did not improve patients’ quality of life
CONVINCE^69^ NCT02898610	Phase 3, R, PG, OL trial. 3154 with non-severe, non-cardioembolic IS or high-risk TIA Female: 30% FU: 28 days	Colchicine (0.5 mg orally per day) plus guideline-based usual care vs. usual care only	Composite of first fatal or non-fatal recurrent IS, MI, cardiac arrest, or hospitalization for unstable angina	There was no difference in the primary endpoint in patients on colchicine vs. placebo (HR 0.84; 0.68–1.05; *P* = 0.12) or the rates of serious adverse events in both groups
PARADISE-MI^71^ NCT02924727	Phase 3, R, DB, PG trial. 5661 with STEMI/NSTEMI (76%/24%), LV dysfunction, pulmonary congestion, and ≥1 risk-enhancing factor Female: 24% FU: 23 months	Sacubitril–valsartan (target dose 97/103 mg b.i.d.) vs. ramipril (target dose of 5 mg b.i.d.), in addition to standard treatment	Cardiovascular death or incident HF, whichever occurred first	The incidence of the primary outcome did not differ between the sacubitril/valsartan and the ramipril groups, irrespective of the type of AMI
STOP-or-NOT trial^72^ NCT03374449	OL, R, pragmatic trial. 2222 treated with ACEIs and ARBs for ≥3 months before major non-cardiac surgery Female: 35% FU: 28 days	Continuation vs. discontinuation of ACEIs/ARBs 48 h prior to surgery	Composite of all-cause mortality and major post-operative complications within 28 days after surgery	Discontinuation of ACEIs/ARBs did not increase the rate of all-cause mortality or major post-operative complications. Episodes of hypotension during surgery occurred were more frequent and longer in the continuation strategy

ACEIs, angiotensin-converting enzyme inhibitors; AF, atrial fibrillation; AHF, acute heart failure; aHR, adjusted hazard ratio; AMI, acute myocardial infarction; ARBs, angiotensin receptor blockers; b.i.d., twice daily; CAD, coronary artery disease; CV, cardiovascular; DB; double-blind; DD, double-dummy; DSMB, Data and Safety Monitoring Board; FU, follow-up; HF, heart failure; HFpEF, heart failure with preserved ejection fraction; HR, hazard ratio; IS, ischaemic stroke; ISRCTN, the UK's Clinical Study Registry; i.v., intravenous; LV, left ventricular; LVEF, left ventricular ejection fraction; MI, myocardial infarction; NCT, ClinicalTrials.gov identifier; NSTEMI, non-ST-elevation myocardial infarction; o.d., once daily; OL, open label; P, prospective; PC, placebo-controlled; PCI, percutaneous coronary intervention; PG, parallel group; R, randomized; RBC, red blood cells; STEMI, ST-elevation MI; TIA, transient ischaemic attack; TXA, tranexamic acid.

a In alphabetic order.

b Acronyms of the trials are summarized in Supplementary material online, *Table S1*.

**Table 3:** Drug safety studies

				
Trial design/population	Objective	Main outcome(s)	Results	Reference
Retrospective cohort study in 204 155 Medicare beneficiaries with AF Females: 52.7% FU: 1 year	Serious bleeding risk for new users of apixaban or rivaroxaban treated with diltiazem or metoprolol	Composite of bleeding-related hospitalization and death with recent evidence of bleeding	Patients receiving diltiazem had an increased risk for the primary outcome and its components as compared with patients receiving metoprolol. The risk increases at doses of diltiazem >120 mg/day. There was no change in the risk of ischaemic stroke or systemic embolism or death without recent bleeding	Ray *et al.*^77^
Population-based cohort study using two US claims datasets including 5637 patients with liver cirrhosis and non-valvular AF	Compare the effectiveness and safety of apixaban vs. rivaroxaban and vs. warfarin in patients with cirrhosis and AF	Efficacy (ischaemic stroke or systemic embolism) and safety outcomes (major haemorrhage: intracranial or GI)	Rivaroxaban initiators had higher rates of major haemorrhagic and GI events than apixaban initiators. Warfarin initiators also had significantly higher rates of major haemorrhage than apixaban initiators, particularly haemorrhagic stroke. There was no different rate of ischaemic events or death between apixaban and rivaroxaban or warfarin	Simon et *al.*^78^
Population-based, nested case–control study using the UK Clinical Practice Research Datalink 42 190 cases with major bleeding matched to 1 156 641 controls with incident AF initiating OACs Female: 40% FU: 4.6 years	Whether concomitant use of SSRIs with OACs increases the risk of major bleeding compared with OAC use alone	Incidence rate ratios of hospitalization for bleeding or death due to bleeding	Concomitant use of SSRIs and OACs increased the risk of major bleeding compared with OACs alone (IRR, 1.33; 95% CI, 1.24–1.42); the risk peaked during the initial months of treatment and persisted for up to 6 months	Rahman *et al*.^80^
Nationwide Danish cohort study 51 794 VTE patients initiating OACs between January 2012 to 31 December 2022 Female: 48%	NSAID use in patients on OACs	Hospital-diagnosed bleeding episodes	Patients treated with OACs for VTE had a more than two-fold increased risk of GI, intracranial, and urinary tract, thoracic, and respiratory tract bleeding	Petersen *et al.*^81^
Retrospective matched cohort study using data from a centralized data registry. 710 with T2D and 979 with overweight/obesity Female: 52% FU: 3 years	Semaglutide vs. non-GLP-1RAs to manage either T2D or weight	Cumulative incidence and hazard ratio of NAION	At 36 months, the cumulative incidence of NAION for the semaglutide vs. non-GLP-1RA cohorts was 6.7% and 0.8% (*P* < 0.001)	Hathaway *et al.*^82^
Population-based cohort study using the Swedish Prescribed Drug Registry 55 777 men with previous MI or underwent PCI treated with nitrates; 5710 with nitrates and a PDE5I FU: 5.9 years	Association between PDE5I use and CV outcomes in men with stable CAD treated with nitrates Exposure was defined as at least two filled prescriptions of any PDE5I	All-cause mortality	The use of PDE5I in combination with nitrates was associated with higher all-cause, cardiovascular, and non-cardiovascular death, MI, HF, revascularization, and MACE. Thus, careful patient-centred consideration before prescribing a PDE5I to patients with CVD on nitrates is warranted	Piccini *et al.*^61^ and Sharma et al.^86^
A case–crossover study in 429 612 individuals from nationwide Danish registries Female: 75.8%	Establish an association between first-ever triptan use and ischaemic events	Acute MI, ischaemic or non-specified stroke	Triptan initiation increased the risk of ischaemic stroke, ischaemic/non-specified stroke, and MI. Case patients had a high-risk CV; in patients with low background CV risk, the risk of an ischaemic event after triptan initiation was very low	Petersen *et al.*^85^

AF, atrial fibrillation; CAD, coronary artery disease; CV, cardiovascular; CVD, cardiovascular diseases; FU, follow-up; GI, gastrointestinal; GLP-1RA, glucagon-like peptide-1 receptor agonist; HF, heart failure; IRRs, incidence rate ratios; MACE, major adverse cardiovascular events; MI, myocardial infarction; NAION, non-arteritic anterior ischaemic optic neuropathy; NSAIDs, non-steroidal anti-inflammatory drugs; OACs, oral anticoagulants; PCI, percutaneous coronary intervention; PDE5I, phosphodiesterase-5 inhibitor; SSRI, selective serotonin reuptake inhibitor; T2D, type 2 diabetes; VTE, venous thromboembolism.

## First-in-class drugs

**Figure 1:**
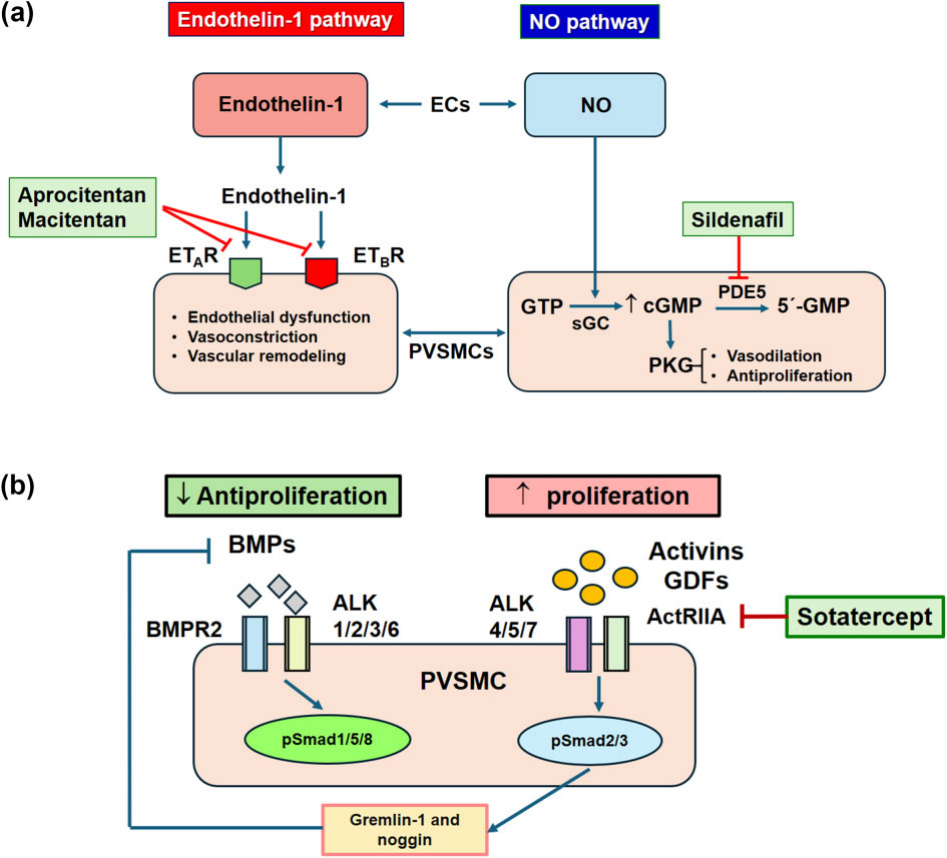
Mechanism of action of first-in-class drugs. (*A*) In pulmonary arterial hypertension (PAH), endothelin-1 (ET-1) activates ET_A_ and ET_B_ receptors leading to endothelial dysfunction, vasoconstriction, vascular remodelling (hypertrophy, fibrosis, and proliferation), inflammation, sympathetic activation, and increased aldosterone synthesis. Aprocitentan, the active metabolite of macitentan, prevents the binding of ET-1 to both ET_A_/ET_B_ receptors and inhibits ET-1-mediated signalling. (*B*) The fixed-dose combination of macitentan, a dual ET_A_ and ET_B_ receptor antagonist, and sildenafil, a phosphodiesterase-5 inhibitor (PDEI), simultaneously targeted the ET-1 and the nitric oxide (NO) pathways involved in the pathophysiology of PAH. NO produced in endothelial cells (ECs) enter the pulmonary vascular smooth muscle cells (PVSMCs) binds to soluble guanylate cyclase (sGC) increasing its catalytic activity, and the conversion of guanosine-5′-triphosphate (GTP) into guanosine 3′,5′-cyclic monophosphate (cGMP), which causes smooth muscle relaxation and antiproliferative effects. This signalling pathway is counterbalanced by sildenafil, which blocks the degradation of cGMP to 5′-guanosine monophosphate. The simultaneous blockade of the ET-1 and NO signalling with macitentan and sildenafil allows to reach better outcomes in patients with PAH as compared with each drug in monotherapy. (*C*) Sotatercept rebalances growth-promoting and growth-inhibiting signalling. In PAH there is a down-regulation of the bone morphogenetic protein (BMP) receptor type II (BMPR2)–Smad1/5/8 pathway in PVSMCs leading to an increased production of activin ligands and an up-regulation of the activin receptor type IIA (ActRIIA)–Smad2/3 pathway. Increased phosphorylated Smad (pSmad)2/3 activity promotes the expression of endogenous BMP antagonists, gremlin-1 and noggin, which further reduces BMP–Smad1/5/8 signalling. The result is a reduction in antiproliferative and an increase in proproliferative signalling pathways counteracting pulmonary vascular remodelling. Sotatercept acts to sequester excess of ActRIIA ligands, thereby reducing ActRIIA–Smad2/3 signalling to rebalance growth-promoting and growth-inhibiting signalling. ALK, activin receptor-like kinase; pSmad, phosphorylated Smad; Smad, small mothers against decapentaplegic protein.

The FDA awards the ‘first-in-class’ designation to products that ‘use a novel and unique mechanism of action to treat a medical condition’.^1^ We believe that aprocitentan, sotatercept, and the first once-daily, single-pill combination containing macitentan and tadalafil (M/T-FDC) for the treatment of PAH represent a significant advance in CV pharmacotherapy. *Figure 1* shows the mechanism of action of these drugs.

1. Aprocitentan, the active metabolite of macitentan, is the first dual ET-1 ET_A_ and ET_B_ receptor antagonist (potency ratio 1:16) approved for uncontrolled hypertension^2^ (*Figure 1A*). After oral administration, it reaches its peak plasma levels within 4–5 h, highly binds to (>99%) plasma proteins, is metabolized by non-cytochrome (CY)P450 enzymes, and presents a half-life of ∼41 h. The clinical development of aprocitentan was based on two trials. A phase 2 dose-finding study in patients with essential hypertension showed that aprocitentan [5—50 mg once a day (o.d.)] dose dependently decreased sitting systolic/diastolic blood pressure (SBP/DBP) with a maximal reduction at 25 mg (9.9/7.0 mm Hg), while lisinopril (20 mg o.d.) decreased SBP/DBP by 4.8/3.8 mm Hg compared with placebo.^3^ The phase 3 PRECISION trial recruited 730 patients with an SBP of ≥140 mm Hg despite taking standardized background therapy with three antihypertensive drugs, including a diuretic, which were continued throughout the study; patients on β-blockers continued this treatment throughout the study.^4^ Patients were randomized to aprocitentan (12.5 or 25 mg) or placebo during a 4-week double-blind treatment period (part 1); then, patients entered a 32-week single (patient)-blind period (part 2) where received aprocitentan 25 mg; finally, patients were re-randomized to aprocitentan 25 mg or placebo during a 12-week double-blind withdrawal period (part 3). The primary and key secondary endpoints were changes in unattended office SBP from baseline to week 4 and from withdrawal baseline to week 40, respectively. At 4 weeks, the least square mean (SE) change in office SBP as compared with placebo was −3.8 mm Hg (−6.8 to −0.8 mm Hg; *P* = 0.0042) for aprocitentan 12.5 mg and the respective difference for 24 h ambulatory SBP −4.3 mm Hg, respectively. The SBP/DBP reduction was maintained for 44 weeks and after 4 weeks of withdrawal, and office SBP significantly increased with placebo vs. aprocitentan (5.8 mm Hg; *P* < 0.001). The 25 mg dose did not produce any further BP reduction as compared with the 12.5 mg, but increased the risk of oedema/fluid retention. Thus, aprocitentan (12.5 mg o.d.) was approved in combination with other antihypertensive drugs, to lower BP in adult patients who are not adequately controlled on other drugs. Aprocitentan increases serum aminotransferase and total bilirubin levels and decreases haemoglobin concentration and haematocrit. It is not recommended in patients with an estimated glomerular filtration rate (eGFR) <15 mL/min, elevated aminotransferases (>3 × the upper limit of normal), or moderate-severe hepatic impairment and is contraindicated during pregnancy. Considering the limited reduction of BP, significant side effects (e.g. oedema), and the very high costs, the clinical added value of aprocitentan requires further clinical investigation.
2. The 2022 European Society of Cardiology and the European Respiratory Society guidelines strongly recommended the initial oral drug combination therapy with a dual ET-1 receptor antagonist (ambrisentan or macitentan) and a phosphodiesterase-5 inhibitor (PDE5I: tadalafil) for patients with idiopathic, heritable, or drug-associated PAH without CV lung complications.^5^ Macitentan counteracts the adverse effects of the abnormal activation of the ET system, reducing pulmonary arterial vasoconstriction, smooth muscle cell proliferation and fibrosis, and the risk of disease progression and hospitalizations, while tadalafil improves exercise ability (*Figure 1A*). The first once-daily, orally active, single-pill combination of macitentan (10 mg) and tadalafil (40 mg) was approved for chronic treatment of PAH [World Health Organization (WHO) Group I] in adult patients of WHO functional class II and III. The approval of the M/T-FDC was based on the results of the A DUE trial, which compared the efficacy and safety of M/T-FDC to each monotherapy, macitentan or tadalafil, in 187 patients with PAH (WHO functional class II and III), including treatment-naïve or previously treated with an ERA or a PDE5I for ≥3 months.^6^ After 16 weeks, M/T-FDC produced a greater pulmonary vascular resistance (PVR) reduction vs. macitentan (geometric mean ratio 0.71; 95% CI 0.61–0.82; *P* < 0.0001) and vs. tadalafil (0.72; 0.64–0.80; *P* < 0.0001) both in treatment-naïve and baseline-treated PAH patients. The use of M/T-FDC reduces pill burden and may improve adherence. Pre-defined adverse events of special interest (oedema/fluid retention, anaemia, and hypotension), but not hepatic disorders, were more common with M/T-FDC. It is contraindicated in patients on nitrates, guanylate cyclase inhibitors (riociguat), or strong CYP3A4 inducers/inhibitors and because it may cause fatal harm, females of reproductive potential must use effective contraception during and for 1 month after treatment with M/T-FDC.
3. Sotatercept is a fusion protein comprising the extracellular domain of the human activin receptor type IIA and the Fc domain of human immunoglobulin G1 (IgG1). It acts as a ligand trap for members of the transforming growth factor-beta (TGF-β) superfamily, thus improving the balance between pro- and antiproliferative signalling to regulate vascular cell proliferation underlying PAH^7^ (*Figure 1B*). It presents an absolute bioavailability of ∼66% and a half-life of ∼24 days and is metabolized into small peptides. Its approval was based on the STELLAR trial, which compared sotatercept (0.3–0.7 mg/kg) vs. placebo administered subcutaneously (s.c.) every 3 weeks on top of background therapies in 323 patients with PAH (WHO functional class II or III).^8^ At 24 weeks, sotatercept increased the baseline 6-min walking distance (6MWD) 41 m (28–54; *P* < 0.001). Time to first occurrence of death or non-fatal clinical worsening events was 84% lower with sotatercept than with placebo [hazard ratio (HR) 0.16; 0.08–0.35; *P* < 0.001]. Significant improvements were also observed in PVR, WHO functional class, N-terminal pro B-type natriuretic peptide (NT-proBNP) levels, French risk model, and PAH-SYMPACT questionnaire. Thus, sotatercept is approved for the treatment of adults with PAH (WHO Group 1) to increase exercise capacity, improve WHO functional class, and reduce the risk of clinical worsening events. Adverse events of sotatercept include headache, erythrocytosis, thrombocytopenia, bleeding, telangiectasia, and increased blood pressure. It may cause foetal harm and women of reproductive potential should be advised to use an effective contraception during treatment and for at least 4 months after the final dose.

## Drugs receiving a new label extension

Once a drug has been approved to treat a given CVD, the results of RCTs may demonstrate that it can be effective for treating an additional disease leading to a label extension. This was the case for two drugs:

1. Semaglutide, a glucagon-like peptide-1 (GLP-1) receptor agonist, is the first drug approved for reducing CV risk in patients with CVD who are either overweight or obese, with or without type 2 diabetes (T2D). This approval was based on the SELECT trial recruiting 17 604 adults with established CVD, and a body mass index (BMI) ≥27 kg/m^2^ without diabetes.^9^ At 39-month follow-up, semaglutide (2.4 mg s.c. once-weekly) was superior to placebo to reduce the composite of CV death, non-fatal myocardial infarction (MI), or non-fatal stroke (HR 0.80; 0.72–0.90; *P* < 0.001) and body weight (—9.39% vs.—0.88%). The effects of semaglutide occurred early after initiation of treatment and were similar across CV endpoints and among pre-specified patient subgroups. Secondary subanalyses pointed to non-significant trends towards benefit for CV death (0.85; 0.71–1.01), heart failure (HF) hospitalization or urgent medical visits (0.82; 0.71–0.96), and all-cause mortality (0.81; 0.71–0.93). Adverse events leading to permanent drug discontinuation occurred more frequently with semaglutide than with placebo (16.6% vs. 8.2%; *P* < 0.001).
2. Bempedoic acid is an ATP citrate lyase inhibitor that targets cholesterol synthesis upstream of 3-hydroxy-3-methylglutaryl coenzyme A reductase, the enzyme inhibited by statins. The label of bempedoic acid, with or without ezetimibe, has been expanded to reduce the risk for MI and coronary revascularization in adults with established CVD or high risk for a CVD event who are unable to take recommended statin therapy (including those not taking a statin). Bempedoic acid (± ezetimibe) indication for lowering LDL cholesterol (LDL-C) has been expanded to all adults with primary hyperlipidaemia, not just those with heterozygous familial hypercholesterolaemia or established atherosclerotic CVD (ASCVD) requiring additional LDL-C reduction. Thus, bempedoic acid (± ezetimibe) is the first oral non-statin LDL-C-lowering drug approved to reduce the risk of CV events in both primary and secondary prevention. This new labelling is based on the CLEAR Outcomes trial recruiting almost 13 970 statin-intolerant patients, with or at high risk for CVD.^10^ After 40.6 months of follow-up, bempedoic acid (± ezetimibe) significantly reduced LDL-C (21%), high-sensitivity C-reactive protein (22%), and the time to first occurrence of a four-component major adverse CV events (MACE-4), composite of CV death, non-fatal stroke, non-fatal MI or coronary revascularization (HR 0.87; 0.79–0.96; *P* = 0.004), fatal or non-fatal MI (0.77; 0.66–0.91; *P* = 0.002), coronary revascularization (0.81; 0.72–0.92; *P* = 0.001), and time to first occurrence of three-component MACE (MACE-3), composite of CV death, non-fatal stroke, or non-fatal MI (0.85; 0.76–0.96; *P* = 0.006).

A pre-specified subanalysis of the CLEAR Outcomes trial evaluated the CV benefits of bempedoic acid in patients with and without T2D, baseline LDL-C ≥2.59 mmol/L, and unable or unwilling to take guideline-recommended doses of statins.^11^ After 3.4 years of follow-up, bempedoic acid monotherapy compared with placebo reduced the incidence of MACE-4 (HR 0.83; 0.72–0.95) and MACE-3 (0.80; 0.68–0.93) in patients with T2D, with similar trends in patients with pre-diabetes and normoglycaemia, although the difference in absolute risk was greatest among patients with T2D (−2.4% for MACE-4, *P*_interaction_ = 0.0063; −2.1% for MACE-3, *P*_interaction_ = 0.010). Among patients without T2D, bempedoic acid did not increase the risk of new-onset diabetes, HbA1c or blood glucose concentrations, and produced a modest weight loss. Placebo-corrected LDL-C, non-HDL cholesterol, and high sensitivity C-reactive protein levels at 6 months were reduced in each glycaemic stratum (diabetes, pre-diabetes, and normoglycaemia) among patients randomized to bempedoic acid (all *P* < 0.001). Thus, in patients with or without T2D unwilling or unable to take guideline-recommended doses of statins, bempedoic acid monotherapy significantly reduced CV events.

## Clinical trials with positive results

### Lipid-lowering drugs

Because older people are underrepresented in clinical trials, the FAST-MI investigators analysed the association between the use and intensity of lipid-lowering therapy (LLT: atorvastatin ≥40 mg or equivalent, or any combination of statin and ezetimibe) in 2258 patients aged ≥80 years admitted for acute MI [AMI: 39% with ST-elevation MI (STEMI), 58% with percutaneous coronary intervention (PCI)] ≤48 h from onset.^12^ Patients were discharged without, with conventional doses, or with high-dose LLT. High-dose LLT was associated with lower 5-year mortality [adjusted hazard ratio (aHR) 0.78; 0.66–0.92] compared with patients without LLT, but not conventional-intensity LLT (aHR 0.93; 0.80–1.09). In propensity score-matched cohorts, 5-year survival was higher in patients receiving high-intensity LLT than in patients without statins at discharge (52% vs. 42%; HR 0.78; 0.62–0.98). These results strongly suggest that high-intensity LLT should not be denied to older patients.

VICTORION-INITIATE compared an ‘inclisiran first’ strategy (adding inclisiran immediately upon failure to reach LDL-C <70 mg/dL despite maximally tolerated statins) with usual care (at the treating provider's judgement) in patients with ASCVD and LDL-C ≥70 mg/dL (mean baseline LDL-C 97.4 mg/dL) or non-HDL cholesterol (non-HDL-C) ≥100 mg/dL, and fasting triglycerides <500 mg/dL.^13^ The ‘inclisiran first’ strategy produced a greater reduction in LDL-C (60.0% vs. 7.0%; *P* < 0.001) and more patients achieved LDL-C levels <70 and <55 mg/dL than in the usual care group (81.8% vs. 22.2% and 71.6% vs. 8.9%; both *P* < 0.001). In the usual care arm fewer patients had LLT intensified and non-statin LLT was underutilized, resulting in underachievement of LDL-C goals. Additionally, statin discontinuation rates (a co-primary endpoint) with ‘inclisiran first’ (6.0%) were non-inferior to usual care (16.7%). Thus, starting inclisiran immediately upon failure to reach LDL-C goals on maximally tolerated statins in patients with ASCVD allows to achieve guideline-recommended LDL-C levels without major safety concerns.

A pooled analysis of data from the Microbleeds International Collaborative Network investigated the association between statin use and the risk of intracranial haemorrhage (ICrH) following an ischaemic stroke (IS) or transient ischaemic attack (TIA) in patients with availability of appropriate baseline magnetic resonance imaging for cerebral microbleed (CMB) quantification and distribution, registration of statin use after the index stroke, and collection of stroke event data during a follow-up period of ≥3 months.^14^ Among 16 373 patients, 10 812 received statins at discharge and 4668 had ≥1 CMBs. Compared with non-users, statin therapy reduced the risk of any stroke (aHR 0.68; 0.56–0.84) and IS (0.65; 0.51–0.82), without increasing the risk of ICrH. Results remained consistent when considering anatomical distribution and the high burden (≥5) of CMBs. Thus, in patients with IS or TIA and CMBs, statins reduced the risk of any stroke or IS without an increased risk of ICrH.

### Antithrombotic drugs and antidotes

Subclinical atrial tachyarrhythmias are independently associated with a 2.5-fold increase in the risk of IS or systemic embolism (IS/SE).^15^ The ARTESIA trial showed that among patients with device-detected subclinical atrial fibrillation (AF) by an implanted pacemaker, defibrillator, or cardiac monitor lasting from 6 min to 24 h (mean CHA2DS2-VASc score 3.9), apixaban as compared with aspirin resulted in a lower risk of IS/SE than aspirin (0.78% vs. 1.24%; HR 0.63; 0.45–0.88; *P* = 0.007), IS (including stroke of unknown cause; 0.62; 0.43–0.91), and stroke from any cause (0.64; 0.46–0.90). However, major bleeding occurred more often with apixaban than with aspirin (1.71% vs. 0.94% per patient-year; HR 1.80; 1.26–2.57; absolute risk increase 0.77%, *P* = 0.001).^16^ Thus, in patients with subclinical AF and increased risk for stroke, the absolute reduction in IS/SE produced by apixaban was lower than the absolute increase of major bleeding (NNT 217; NNH 129). The 2024 ESC Guidelines for the management of AF recommend that oral anticoagulant (OAC) therapy may be considered in patients with asymptomatic device-detected subclinical AF and elevated thrombo-embolic risk to prevent IS and thromboembolism, excluding patients at high risk of bleeding (class IIb, level B).^17^

Although clinical guidelines consistently recommend the use of monotherapy with OACs after the early period of dual antithrombotic treatment, data on long-term antithrombotic strategy for patients with AF and stable coronary artery disease (CAD) are limited. The EPIC-CAD trial compared edoxaban monotherapy (60 mg o.d.; 30 mg o.d. with CrCl 15–50 mL/min, body weight ≤60 kg, and use of certain P-glycoprotein inhibitors) with dual antithrombotic therapy (edoxaban plus a single antiplatelet agent) in 554 patients with AF (mean CHA_2_DS_2_-VASc score 4.3) and stable CAD. At 12 months, edoxaban monotherapy reduced the risk of a composite of all-cause mortality, MI, stroke, SE, unplanned urgent revascularization, or major or clinically relevant non-major bleeding as compared with dual antithrombotic therapy (HR 0.44; 0.30–0.65; *P* < 0.001).^18^ This result appeared to be driven mainly by a lower incidence of major or clinically relevant non-major bleeding events (HR 0.34; 0.22–0.53), while the risk of ischaemic events and mortality was similar in both groups.

Clinical guidelines recommend dual antiplatelet therapy (DAPT) with aspirin plus a P2Y12 receptor inhibitor for 12 months following PCI to prevent MI and stent thrombosis.^19^ In patients with acute coronary syndrome (ACS), unguided de-escalation from DAPT to P2Y12 inhibitor monotherapy after PCI was associated with the lowest risk of MACE and bleeding outcomes, showing the lack of usefulness of platelet function testing,^19^ while short DAPT followed by P2Y_12_ inhibitor was associated with the lowest risk of major bleeding and all-cause mortality.^20^ However, no head-to-head comparisons exist between all different single antiplatelet monotherapies post-ACS. The ULTIMATE-DAPT trial randomized participants who completed the IVUS-ACS study without major ischaemic or bleeding events after 1-month treatment to DAPT (oral ticagrelor, 90 mg b.i.d., plus aspirin 100 mg o.d.) or oral ticagrelor plus placebo, beginning 1 month and ending at 12 months after PCI. In 3399 patients, 1-month DAPT (ticagrelor plus aspirin) followed by ticagrelor monotherapy reduced clinically relevant bleeding (2.1% vs.4.6%; HR 0.45; 0.30–0.66; *P* < 0.0001), but provided a similar protection from major adverse CV or cerebrovascular events (MACE: composite of cardiac death, MI, IS, definite stent thrombosis, or clinically driven target vessel revascularization) as compared with DAPT (0.98; 0.69–1.39; *P*_non__-__inferiority_ < 0.0001, *P*_superiority_ = 0.89).^21^ The main limitations of this study are that the primary efficacy endpoint included minor bleeding [Bleeding Academic Research Consortium (BARC) 2-type], it was largely underpowered to assess efficacy (predicted number of events in the control arm: 6.2%, observed 3.7%), efficacy included un-conventional and soft endpoints (e.g. definite stent thrombosis, or clinically driven target vessel revascularization) rather than MACE-4, and the lack of aspirin-only arm for comparison.

The OPT-BIRISK trial was designed to compare clopidogrel monotherapy with DAPT with clopidogrel and aspirin as maintenance therapy for an additional 9 months in 7700 ACS patients deemed at both thrombotic and bleeding risks who completed 9–12 months of DAPT following drug-eluting stent (DES) PCI. As compared with DAPT, clopidogrel monotherapy reduced the risk of BARC types 2, 3, or 5 bleeding (2.5% vs. 3.3%; HR 0.75; 0.57–0.97; *P* = 0.03), even though the observed number of events was approximately half than those pre-specified in sample size determination (predicted 6%, observed 3.3%) and the difference was driven only by BARC 2 bleeding, with no difference for BARC 3 and 5 bleeding.^22^ Efficacy endpoint (a composite of all-cause mortality, MI, stroke, and clinically driven revascularization) was only a secondary outcome, expected to occur in 8%, but observed in 3.5% and 2.6% in the DAPT and clopidogrel arms, respectively (0.74; 0.57–0.96; *P*_non__-__inferiority_ < 0.001; *P*_superiority_ = 0.02), and the difference was mainly driven by revascularization.

Given the limitations of the above trials, more robust data and rigorous trial designs are needed to assess the efficacy and duration of a shorter DAPT (1, 3, and 6 months?) as well as the most effective type (aspirin alone?, clopidogrel, prasugrel, or ticagrelor alone?) of single antiplatelet therapy post-DAPT with the last generation of less thrombogenic stents.

The ADALA trial compared in 90 patients (53 patients with previous major bleeding events) the efficacy and safety of low-dose DOAC (apixaban 2.5 and 5 mg b.i.d.) vs. DAPT (100 mg aspirin plus clopidogrel 75 mg o.d.) after successful percutaneous left atrial appendage occlusion (LAAO).^23^ At 3 months, compared with DAPT, low-dose DOAC reduced the primary endpoint [composite of major bleeding and thrombo-embolic events including stroke, SE, and device-related thrombosis (DRT)] (4.5% vs. 21.7%; HR 0.19; *P* = 0.02) and the rate of DRT (0% vs. 8.7%; *P* = 0.04), but there were no differences in major bleeding or thrombo-embolic events between groups. Thus, low-dose DOAC for 3 months after LAAO was associated with a better efficacy and safety balance compared with DAPT. However, these results will need to be confirmed in future larger RCTs.

The DACAB trial found that among 500 patients undergoing elective coronary artery bypass grafting surgery (CABG), graft patency (primary outcome) at 1 year was significantly higher in patients randomized to DAPT compared with patients randomized to aspirin alone (88.7% vs. 76.5%; *P* < 0.001).^24^ Because this trial was not powered for clinical outcomes, the follow-up of patients included in the DACAB trial was extended to 5 years.^25^ At the end of the study, the risk of MACE was lower with DAPT vs. aspirin monotherapy (22.6% vs. 29.9%; HR 0.65; 0.43–0.99; *P* = 0.04) and vs. ticagrelor monotherapy (0.66, 0.44–1.00; *P* = 0.05). Results were consistent across key clinical subgroups, including different baseline bleeding risks. Thus, ticagrelor DAPT for 1 year after surgery reduced the risk of MACE at 5 years after CABG compared with aspirin or ticagrelor monotherapy.

The T-PASS trial investigated whether ticagrelor monotherapy after <1 month of DAPT was non-inferior to 12 months of ticagrelor-based DAPT in 2850 patients with ACS who underwent DES PCI.^26^ Aspirin was discontinued at a median of 16 days (12–25 days) in patients receiving ticagrelor monotherapy. Stopping aspirin within 1 month followed by ticagrelor monotherapy was both non-inferior and superior to 12-month DAPT for the 1-year composite of all-cause mortality, MI, definite or probable stent thrombosis, stroke, and BARC 3–5 bleeding (HR 0.54; 0.37–0.80; *P* < 0.001 for non-inferiority; *P* = 0.002 for superiority), primarily driven by a significant reduction in BARC 3–5 bleeding (1.2% vs. 3.4%; 0.35; 0.20–0.61; *P* < 0.001). The main limitations include the open-label not placebo-controlled design, the lower observed (5.2%) than anticipated (14%) events rates suggesting inclusion of non-high-risk patients, the involvement of Korean patients only, and the implantation of biodegradable polymer sirolimus-eluting stents. Thus, the results may not extend to other populations at high risk or to patients who receive other stent types, and might not be directly translated to other geographic regions.

The ANNEXA trial compared andexanet alfa, a modified recombinant inactive form of human factor Xa, which binds and sequesters factor Xa inhibitors, with usual care (85.5% receiving prothrombin complex concentrates) on haematoma volume expansion in patients receiving a factor Xa inhibitor within 15 h before an acute intracerebral haemorrhage.^27^ About 90% of patients had AF, 21% stroke, and 12% MI. Haemostatic efficacy, defined as an expansion of the haematoma volume ≤35% at 12 h, an increase of <7 points on the National Institutes of Health Stroke Scale (NIHSS), and no receipt of rescue therapy between 3 and 12 h, was more often achieved in the andexanet group than in the usual care group (67.0% vs. 53.1%; *P* = 0.003). Haematoma volume expansion ≤35% was observed in 76.7% of patients receiving andexanet and in 64.6% of those on usual care. However, thrombotic events (10.3% vs. 5.6%; *P* = 0.048), including IS (6.5% vs.1.5%), were more common in the andexanet group. Multiplicity-unadjusted results for death and a good outcome on the modified Rankin scale did not differ appreciably between groups, and the trial was not powered to detect differences in these outcomes. Thus, further studies are needed to define the potential net benefit of andexanet as an antidote in patients receiving anti-Xa DOACs.

### Cardiovascular and renal effects of semaglutide

Semaglutide is a glucagon-like peptide-1 receptor agonist (GLP-1RA) that stimulates glucose-dependent insulin secretion, reduces glucagon release and hepatic gluconeogenesis, and reduces food intake by slowing gastric emptying and promoting satiety.^28^ A pre-specified analysis of the SELECT trial compared the effect of semaglutide with placebo in 4286 patients with ASCVD and overweight or obesity and a history of HF [with preserved (HFpEF) or reduced ejection fraction (HFrEF), or unclassified].^29^ In patients with HF as compared with those without HF, semaglutide reduced MACE (composite of non-fatal MI, non-fatal stroke, and CV death; HR 0.72; 0.60.0.87), HF composite endpoints (CV death or hospitalization or urgent hospital visit for HF: 0.79; 0.64–0.98), CV death (0.76; 0.59–0.97), and all-cause mortality (0.81; 0.66–1.00). Semaglutide improved outcomes early after initiation of therapy in patients with HFrEF (MACE: 0.65; 0.49–0.87; composite HF endpoint: 0.79, 0.58–1.08) and HFpEF (0.69; 0.51–0.91 and 0.75; 0.52–1.07, respectively) and the benefit persisted over the trial period being independent of age, sex, BMI, New York Heart Association (NYHA) status, and diuretic use. Discontinuation rates for semaglutide were similar in patients with and without HF. Thus, in overweight or obese patients with ASCVD, but not T2D, semaglutide reduced MACE, HF composite endpoints and CV, and all-cause mortality in patients with HF, regardless of HF subtype.

Diabetes is the leading cause of chronic kidney disease (CKD), and patients with T2D and CKD are at high risk for HF and premature CV death.^30^ The FLOW trial randomized patients with T2D and CKD [defined by an eGFR of 50–75 mL/min/1.73 m^2^ and a urinary albumin-to-creatinine ratio (UACR) of >300 and <5000 mg/g, or an eGFR of 25 to <50 mL/min/1.73 m^2^, and a UACR >100 and <5000 mg/g] to semaglutide (1 mg s.c. weekly) or placebo.^31^ At 3.4 years, semaglutide reduced the risk of major kidney disease events (HR 0.76; 0.66–0.88; *P* = 0.0003), kidney-specific components of the primary outcome (0.79; 0.66–0.94), and CV death (0.71; 0.56–0.89) vs. placebo. Secondary outcomes also favoured semaglutide, including mean annual eGFR slope (−2.19 vs. −3.36 mL/min/1.73 m^2^ per year; *P* < 0.001), risk of MACE (0.82; 0.68–0.98; *P* = 0.029), all-cause mortality (0.80; 0.67–0.95, *P* = 0.01), and serious adverse events. Thus, in patients with T2D and CKD, semaglutide reduced the risk of major kidney disease events and CV death. In a pre-specified analysis of this trial, semaglutide compared with placebo increased the time to first HF events or CV death (HR 0.73; 0.62–0.87; *P* = 0.0005), HF events alone (0.73; 0.58–0.92; *P* = 0.0068), and CV death alone (HR 0.71; 0.56–0.89; *P* = 0.0036).^32^ The risk reduction for the composite HF outcome was similar in patients with (0.73; 0.54–0.98; *P* = 0.03) and without HF at baseline (0.72; 0.58–0.89; *P* = 0.002) and across a range of clinically relevant subgroups.

HFpEF is associated with a high risk of hospitalization and death, especially in patients who are overweight, obese, or have T2D. The STEP-HFpEF DM randomized 616 patients with HFpEF, BMI ≥30 kg/m², and T2D to semaglutide (2.4 mg s.c. once weekly) or placebo. After 52 weeks, semaglutide improved quality-of-life parameters evaluated by the Kansas City Cardiomyopathy Questionnaire Clinical Summary Score (KCCQ-CSS) (estimated difference 7.3 points; 4.1–10.4; *P* < 0.001) and reduced body weight (estimated difference, −6.4 percentage points; −7.6 to −5.2; *P* < 0.001).^33^ The results for all the confirmatory secondary endpoints (estimated between-group difference in change in 6MWD; win ratio for a hierarchical composite endpoint that included death, HF events, and differences in the change in the KCCQ-CSS and 6MWD; and estimated treatment ratio for change in C-reactive protein level) favoured semaglutide over placebo (all *P* < 0.001). These results, together with those of the STEP-HFpEF trial,^34^ confirmed that among patients with obesity-related HFpEF and T2D, semaglutide (2.4 mg s.c. once weekly) significantly reduced HF-related symptoms and physical limitations.

Because of the relatively modest size of both trials, several pre-specified pooled analyses of individual patient data from the STEP-HFpEF and STEP-HFpEF DM trials were performed to provide a more definitive assessment of the efficacy and safety of semaglutide. (1) In one analysis, semaglutide compared with placebo significantly improved the KCCQ-CSS (mean difference 7.5 points), 6MWD (mean between-group difference 17.1 m), and the hierarchical composite endpoint (win ratio 1.65) and reduced C-reactive protein levels and body weight compared with placebo (all *P* < 0.0001) and its efficacy was consistent across multiple subgroups.^35^ (2) Another secondary analysis evaluated whether the effects of semaglutide differ in patients with and without AF (and across various AF types).^36^ Semaglutide led to larger improvements in the KCCQ-CSS (11.5 vs. 4.3 points; *P*_interaction_ = 0.001) and the hierarchal composite endpoint (win ratio of 2.25 vs. 1.30; *P*_interaction_ < 0.001) in participants with AF vs. without AF, respectively. Semaglutide also significantly improved HF-related symptoms, physical limitations, and exercise function and reduced body weight, C-reactive protein, and NT-proBNP levels regardless of AF status. Thus, semaglutide-mediated improvements in HF-related symptoms and physical limitations were more pronounced in those with vs. without AF at baseline. (3) Another pooled analysis examined whether efficacy and safety endpoints differed by baseline diuretic use, and the effect of semaglutide on loop diuretic use and dose over the 52-week treatment period.^37^ As compared with placebo, in semaglutide-treated patients loop diuretic dose decreased by ∼20%, were more likely to experience a loop diuretic dose reduction, and less likely to experience a dose increase (all *P* < 0.0001). Reductions in body weight and improvements in exercise function with semaglutide vs. placebo were consistent in all diuretic use categories. These results support a primary decongestive effect of semaglutide in obesity-related HFpEF.

Another pooled analysis of individual patient-level data from the SELECT, FLOW, STEP-HFpEF, and STEP-HFpEF DM evaluated whether semaglutide reduces clinical HF events.^38^ STEP-HFpEF and STEP-HFpF DM trials enrolled patients with obesity-related HFpEF, SELECT trial patients with ASCVD and overweight or obesity but without T2D, and FLOW trial patients with T2D and CKD. Participants were randomized to once-weekly s.c. semaglutide (2.4 mg in the STEP-HFpEF, STEP-HFpEF DM, and SELECT; 1.0 mg in the FLOW). Compared with placebo, semaglutide reduced the risk of the composite endpoint of HF events (hospitalization or urgent visit due to HF) or CV death (HR 0.69; 0.53–0.89; *P* = 0.0045) and the risk of worsening HF events alone (0.59; 0.41–0.82; *P* = 0.0019), but not CV death (0.82; 0.57–1-16). These data support the use of semaglutide to reduce the risk of HF events in patients with HFpEF.

### Tirzepatide vs. semaglutide

A propensity-matched, cohort study identified individuals with overweight or obese who initiated tirzepatide or semaglutide labelled for T2D using electronic health record data.^39^ Patients receiving tirzepatide were significantly more likely to achieve weight loss ≥5% (HR 1.76; 1.68–1.84), ≥10% (2.54; 2.37–2.73), and ≥15% (3.24; 2.91–3.61) than those using semaglutide, and on-treatment changes in weight were larger for patients receiving tirzepatide at 3 (5.9% vs. 3.6%), 6 (10.1% vs. 5.8%), and 12 months (15.3% vs. 8.3%). After adjusting for residual confounders, the absolute difference in weight loss between tirzepatide and semaglutide was −2.4% (−2.5% to −2.2%), −4.3% (−4.7% to −4.0%), and −6.9% (−7.9% to −5.8%) at 3, 6, and 12 months of treatment, respectively. A weight loss of ≥15% was achieved by 42.3% of the tirzepatide group vs. 18.1% of the semaglutide group. Rates of gastrointestinal adverse effects were similar between groups and 53% discontinued the medication during the study. Future RCTs are needed to compare the effect of tirzepatide and semaglutide on MACE.

### Sodium–glucose cotransporter-2 inhibitors

The EMPA-KIDNEY trial showed that among patients with CKD at risk of disease progression [i.e. sustained eGFR decline ≥40% from randomization, end-stage kidney disease (ESKD), sustained eGFR <10 mL/min/1.73 m², or kidney failure death], empagliflozin reduced the risk of kidney disease progression or CV death (HR 0.72; 0.64–0.82; *P* < 0.001) compared with placebo.^40^ The trial was stopped prematurely after a median follow-up time of 2 years, but 4891 patients (74%) originally randomized were enrolled for another 2 years to assess how the effects of empagliflozin evolve after drug discontinuation.^41^ During this period, local practitioners could prescribe open-label sodium–glucose cotransporter-2 inhibitors (SGLT2Is), including open-label empagliflozin, but the use of SGLT2Is was similar in the empagliflozin and placebo groups (43% vs. 40%). During the entire follow-up period (i.e. active trial plus post-trial observation periods), empagliflozin, as compared with placebo, reduced: (1) the primary endpoint of kidney disease progression or CV death (HR 0.79; 0.72–0.87); (2) the risks of kidney disease progression (0.79; 0.72–0.87), all-cause mortality or ESKD (0.81; 0.72–0.90), and ESKD (0.74; 95% CI 0.64–0.87); and (3) the risk of CV death (0.75; 0.59–0.95), but not the risk of non-CV death (5.3% vs. 5.3%; 0.97; 0.79–1.20). These results confirmed that 2-year treatment with empagliflozin had residual cardiorenal benefits over placebo, for up to 12 months after drug discontinuation.

### Colchicine

Colchicine is approved to reduce CV risk in patients with CAD, but limited data are available about its impact on coronary plaque stability. The COLOCT trial^42^ randomized 128 patients with ACS with lipid-rich plaque (lipid pool arc >90°) to colchicine (0.5 mg o.d.) or placebo. After 12 months, compared with placebo, colchicine increased minimal fibrous cap thickness (51.9 vs. 87.2 μm; *P* = 0.006) and reduced average lipid arc (−25.2° vs. −35.7°; *P* = 0.004), mean angular extension of macrophages (−8.9° vs. −14.0°; *P* = 0.044), high-sensitivity C-reactive protein (*P* = 0.046), interleukin-6 (*P* = 0.025), and myeloperoxidase levels (*P* = 0.047). Thus, it improved coronary plaque stability as assessed by optical coherence tomography in patients with ACS. Because patients with T2D present a higher risk of CV events post-MI partly due to vascular inflammation, it was hypothesized that they could benefit from colchicine use. In a pre-specified subgroup analysis of the COLCOT trial enrolling 959 patients with T2D and recent MI, colchicine reduced the risk of a composite of CV death, resuscitated cardiac arrest, MI, stroke, or urgent hospitalization for angina requiring coronary revascularization (HR 0.65; 0.44–0.96; *P* = 0.03), but increased the incidence of nausea and pneumonia (2.4% vs. 0.4%; *P* = 0.008).^43^

### Cardiomyopathies

Acoramidis is a high-affinity transthyretin (TTR) stabilizer designed to mimic the structural influence of the protective T119M mutation that inhibits the dissociation of tetrameric TTR.^44^ In the ATTRibute-CM trial recruiting 632 patients with transthyretin amyloid cardiomyopathy (ATTR-CM), acoramidis (800 mg b.i.d.) significantly improved the four-step primary hierarchical outcome (all-cause mortality, CV-related hospitalization, and change from baseline in NT-proBNP levels and 6MWD). The corresponding win ratio was 1.8 (1.4–2.2; *P* < 0.0001), with 63.7% of pairwise comparisons favouring acoramidis (35.9% favouring placebo).^45^ The win ratios for the pre-specified secondary analyses performed according to either the two-component hierarchy of all-cause mortality and CV-related hospitalization or the three-component hierarchy of all-cause mortality, CV-related hospitalization, and 6MWD were 1.5 (1.1–2.0) and 1.4 (1.1–1.8) in favour of acoramidis, respectively. Acoramidis also significantly reduced CVD-related hospitalizations (26.7% vs. 42.6%; *P* < 0.0001), but not all-cause mortality. The overall incidence of adverse events was similar in both groups. Thus, acoramidis represents an effective and safe therapeutic option for patients with ATTR-CM.

Patients who completed this study (389 of 632 participants) were invited to enter an open-label extension study. Patients who previously received acoramidis through month 30 continued on acoramidis, and those treated with placebo were switched to acoramidis; participants receiving tafamidis were required to discontinue it.^46^ At month 42, early administration of acoramidis reduced all-cause mortality (HR 0.64; 0.47–0.88; *P* = 0.006), all-cause mortality or first CVD-related hospitalizations (HR 0.57; 0.46–0.72; *P* < 0.0001), and CVD-related hospitalization alone (HR 0.53; 0.41–0.69; *P* < 0.0001) as compared with the placebo. Treatment effects for NT-proBNP, 6MWD, and quality of life assessed by the Kansas City Cardiomyopathy Questionnaire Overall Summary (KCCQ-OS) also favoured acoramidis, and no new clinically important safety issues were identified during the study. Thus, this extended follow-up confirmed the safety of acoramidis.

Vutrisiran is a small-interfering RNA (siRNA) that specifically targets variants and wild-type TTR messenger RNA in the hepatocytes, causes its catalytic degradation, and decreases serum TTR protein levels. In the HELIOS-B trial, patients with ATTR-CM were randomized to vutrisiran or placebo, but 40% of patients received tafamidis at baseline in both groups.^47^ The efficacy endpoints were assessed in patients taking (overall population) or not tafamidis (monotherapy) and tested hierarchically. At 42 months, as compared with placebo, vutrisiran reduced the risk of all-cause mortality and recurrent CV events (overall population: HR 0.72; 0.56–0.93; *P* = 0.01; monotherapy population: 0.67; 0.49–0.93; *P* = 0.02) and all-cause mortality (0.65; 0.46–0.90; *P* = 0.01). In the overall population, vutrisiran resulted in loss of a decline in the 6MWD (least-squares mean difference, 26.5 m; *P* < 0.001) and KCCQ-OS score (least-squares mean difference, 5.8 points; *P* < 0.001) than placebo. Similar benefits were observed in the monotherapy population. The incidence of adverse events was similar in both groups. This is the first study to show the benefit of gene silencers in a cardiomyopathy.

### Heart failure

Finerenone is a non-steroidal mineralocorticoid receptor antagonist (MRA) that reduces the risk of CV outcomes and kidney disease progression in a broad range of patients with CKD and T2D.^48^ The FINEARTS-HF trial tested the hypothesis that finerenone (maximum dose of 20–40 mg o.d.), in addition to usual therapy, would reduce the rate of total worsening HF events and CV death among patients with HF and mildly reduced ejection fraction (EF) (HFmrEF) or HFpEF. At 32 months, finerenone reduced the primary outcome [composite of total worsening HF events (first or recurrent unplanned hospitalizations or urgent visits for HF) and CV death] as compared with usual therapy [relative risk (RR) 0.84; 0.74–0.95; *P* = 0.007] and total number of worsening HF events (0.82; 0.71–0.94; *P* = 0.006), but not CV death.^49^ The results were consistent across all pre-specified subgroups. As expected, finerenone increases serum creatinine and potassium levels (sK^+^) as compared with placebo.

Several pre-specified subanalyses of this trial estimated the long-term effects of finerenone on: (1) *Survival-free from primary end point*. Finerenone extended event-free survival from primary endpoint after 55 years (3.1 years; 0.8–5.4; *P* = 0.007) and after 65 years (2.0 years; 0.8–3.3; *P* = 0.001); these lifetime gains were also observed among patients already treated with SGLT2Is (65-year-old participant: 3.1 years; 0.1–6.0 years; *P* =0.04).^50^ Indeed, finerenone reduced CV death and total HF events in patients treated or not with an SGLT2I at baseline (*P*_interaction_ = 0.76).^51^ (2) *Clinical outcomes based on sK^+^*. Finerenone increases the risk of hyperkalaemia (HR 2.16), but the benefits of finerenone relative to placebo on worsening HF or CV death persisted in patients with sK^+^ >5.5 mmol/L.^52^ These results suggest that dose selection according to baseline eGFR and dose adjustments in response to sK^+^ may mitigate risks of serious hyperkalaemia.

The SUMMIT trial studied the effect of tirzepatide (up to 15 mg s.c. once per week), a dual agonist of glucose-dependent insulinotropic polypeptide (GIP) and GLP-1 receptors, in 731 patients with a BMI ≥30 kg/m^2^ and HFpEF.^53^ After 104 weeks, tirzepatide reduced the composite of adjudicated CV death or a worsening HF event (HR 0.62; 0.41–0.95; *P* = 0.026). The benefit was driven by a reduction in the risk of worsening HF events resulting in hospitalization, intravenous (i.v.) drug therapy in an urgent care setting, or intensification of oral diuretic therapy (0.54; 95% CI 0.34–0.85). HF hospitalizations were also reduced by tirzepatide (0.44; 95% CI 0.22–0.87), but not adjudicated CV death. At 52 weeks, tirzepatide, as compared with placebo, increased the KCCQ-CSS (19.5 vs. 12.7; *P* < 0.001), 6MWD (26.0 vs. 10.1 m; *P* < 0.001), SBP (−4.6 vs. 0.1 mm Hg; *P* < 0.05), high-sensitivity C-reactive protein level (−38.8% vs. −5.9%; *P* < 0.001), and body weight (−13.9% vs. −2.2%; *P* < 0.001). These benefits were consistent across all major subgroups. Adverse events (mainly gastrointestinal) leading to drug discontinuation were more frequently with tirzepatide than with placebo (6.3% vs. 1.4%). Thus, in obese patients with HFpEF, tirzepatide reduced the risk of worsening HF compared with placebo, reduced HF symptom severity, and improved exercise tolerance.

### Intensive vs. standard blood pressure control

Two RCTs analysed whether lowering SBP to <120 mm Hg is superior to that of <140 mm Hg. The BPROAD trial investigated whether intensive treatment targeting an SBP of <120 mm Hg would be more effective than standard treatment targeting an SBP of <140 mm Hg in reducing major CVD events among patients with T2D.^54^ At 1 year of follow-up, mean SBP was 121.6 mm Hg (median, 118.3 mm Hg) in the intensive treatment group and 133.2 mm Hg (median, 135.0 mm Hg) in the standard treatment group. During a median follow-up of 4.2 years, primary outcome events (non-fatal stroke, non-fatal MI, treatment or hospitalization for HF, or CV death) were lower in the intensive treatment group than in the standard treatment group (1.65 vs. 2.09 events per 100 person-years, respectively; HR 0.79; 0.69–0.90; *P* < 0.001). The benefits of intensive treatment were consistent across all pre-specified subgroups. Symptomatic hypotension and hyperkalaemia were significantly more common in the intensive treatment group than in the standard treatment group. Thus, among patients with T2D and an increased risk of CVD, the incidence of MACE was lower with intensive treatment than with standard treatment.

The ESPRIT trial also compared both strategies in patients at high CV risk (38.7% with diabetes, 28.9% with CAD, and 26.9% with stroke).^55^ Mean SBP during follow-up (except the first 3 months) was 119.1 mm Hg in the intensive treatment group and 134.8 mm Hg in the standard treatment group. After 3.4 years of follow-up, the intensive treatment strategy reduced the primary outcome event (composite of CV death, MI, stroke, coronary/non-coronary revascularization, or HF hospitalization) as compared with standard control (9.7% vs. 11.1%; HR 0.88; 0.78–0.99; *P* = 0.028), with no heterogeneity of effects by diabetes status/duration or history of stroke. The risks of all-cause mortality (0.79; 0.64–0.97; *P* = 0.025) and composite of primary outcome or death from any cause (0.89; 0.80–0.99; *P* = 0.039) were lower in the intensive treatment group. There were no significant between-group differences in the serious adverse events of hypotension, electrolyte abnormality, injurious fall, or acute kidney injury, but syncope events occurred more frequently in the intensive treatment group (0.4% vs. 0%). Thus, among Asian hypertensive, intensive SBP control, as compared with standard BP control, prevents major vascular events, with minor excess risk.

## Clinical trials with negative results

### β-Adrenoceptor blockers

AMI guideline recommendations regarding β-blocker utilization for secondary prevention in patients with preserved left ventricular ejection fraction (LVEF; ≥50%) are uncertain,^19^ mainly because trials were conducted years ago, before PCI and modern therapies for MI (antithrombotic agents, high-intensity statins, and renin–angiotensin–aldosterone system inhibitors) were widely used. The REDUCE-AMI trial assigned 5020 patients 1–7 days after MI with preserved LVEF (≥50%) to either long-term β-blocker (metoprolol or bisoprolol) or no β-blocker treatment. After 3.5 years, metoprolol or bisoprolol did not reduce all-cause or CV death, new MI, and hospitalization for AF or HF compared with usual care (HR 0.96; 0.79–1.16; *P* = 0.64),^56^ There were also no differences in safety endpoints, including second-/third-degree atrioventricular block, hypotension, syncope, implantation of a pacemaker or hospitalization for bradycardia, asthma, chronic obstructive pulmonary disease, or stroke. The results of this study, which represent the first modern RCT with β-blockers, suggest that patients with preserved LVEF following MI may not benefit from β-blocker therapy without another indication for their use.

The appropriate duration of treatment with β-blocker drugs after an MI remains unknown, but it is possible that the interruption of long-term treatment may reduce side effects and improve quality of life in patients with a history of uncomplicated MI with an LVEF ≥40% and no other primary indication for β-blocker therapy. To answer this question, the AβYSS trial randomized 3698 patients with a prior MI (63% with STEMI), LVEF ≥40%, and no history of a CV event in the previous 6 months to interruption or continuation of β-blocker treatment.^57^ The median time between the last MI and randomization was 2.9 years, the median follow-up 3.0 years, and the primary endpoint was a composite of death, non-fatal MI, non-fatal stroke, or hospitalization for CV indication. The primary endpoint of non-inferiority of interruption, as compared with continuation, with respect to CV events was not met (HR 1.16; 1.01–1.33; *P*_non__-__inferiority_ = 0.44). Accordingly, there were no statistical differences between the two therapy arms. Additionally, β-blocker interruption did not seem to improve the patients’ quality of life and the trend for increased events in the interruption group was only driven by hospitalization for CV indications, while other endpoint components (death, non-fatal MI, and non-fatal stroke) were identical with both strategies. These results do not support interruption of chronic β-blocker treatment in post-MI patients.

Observational studies suggested that cardioselective β-blockers may decrease the risk of death and exacerbations in patients with chronic obstructive pulmonary disease. The BICS trial investigated whether bisoprolol, a β_1_-selective blocker, reduced the rate of chronic obstructive pulmonary disease exacerbations in patients with chronic obstructive pulmonary disease at high risk of exacerbations.^58^ At 52 weeks, treatment with bisoprolol as compared with placebo did not reduce the number of self-reported chronic obstructive pulmonary disease exacerbations requiring treatment with oral corticosteroids and/or antibiotics [incidence rate ratio (IRR) 0.97; 0.84–1.13; *P* = 0.72) or increase serious adverse events or total or respiratory adverse reactions.

### Antithrombotic drugs

Atrial cardiomyopathy in the absence of AF increases risk of IS, incident AF, and mortality,^59^ but it is uncertain whether anticoagulation is superior to antiplatelet therapy for preventing recurrent cryptogenic stroke. The ARCADIA trial compared apixaban (2.5 or 5 mg b.i.d.) vs. aspirin (81 mg o.d.) for secondary stroke prevention in patients with cryptogenic stroke and atrial cardiomyopathy. After 1.8 years, apixaban did not reduce the risk of recurrent stroke or increased the risk of symptomatic ICrH, other major bleeding, or death compared with aspirin.^60^ Thus, it was recommended to stop further recruitment for futility after a planned interim analysis.

The OCEANIC-AF trial was designed to test the hypothesis that asundexian, a factor XIa inhibitor, would be at least non-inferior to apixaban for the prevention of stroke or SE (S/SE) but superior for bleeding avoidance, providing a net clinical benefit in persons with AF at risk for stroke.^61^ This trial was stopped prematurely when S/SE occurred over three times more in the asundexian group than in the apixaban group (1.3% vs. 0.4%; HR 3.79; 2.46–5.83), although major bleeding occurred in fewer patients treated with asundexian than in those treated with apixaban (0.2% vs. 0.7%; 0.32; 0.18–0.55). Further studies are ongoing to determine whether the concept of factor XIa inhibition may become a target for stroke prevention in patients with AF, reducing bleeding risk as compared with DOACs, and their use for other indications.

Intravenous tranexamic acid (TXA) during cardiac surgery is recommended to reduce bleeding risks. The DEPOSITION trial compared i.v. TXA through surgery or topical TXA at the end of surgery among patients undergoing open cardiac surgery on cardiopulmonary bypass.^62^ Topical TXA did not reduce in-hospital seizures, all-cause mortality, MI, or stroke but increased red blood cell transfusions (35.1% vs. 26.8%; *P* = 0.007) and haemorrhages requiring ≥4 units of red blood cells compared with i.v. TXA (absolute risk increase 8.2%; 5.2–11.5; *P* = 0.007). These results oppose the routine use of topical TXA to control perioperative bleeding in cardiac surgery.

### Lipid-lowering drugs

The AEGIS-II trial found that among 18 219 patients with AMI, multivessel CAD, and additional CV risk factors, four weekly infusions of CSL112 (a human plasma-derived apolipoprotein A1 formulated with phosphatidylcholine) did not reduce the risk of recurrent CV events (MI, stroke, or CV death) at 90 days as compared with placebo (4.8% vs. 5.2%; HR 0.93; 0.81–1.05; *P* = 0.24).^63^ Hypersensitivity or anaphylactoid reactions leading to drug discontinuation were higher in the CSL112 than in the placebo group (*P* = 0.02).

### Sodium–glucose cotransporter-2 inhibitors after MI

The DAPA-MI trial evaluated the effect of dapagliflozin (10 mg o.d.) vs. placebo, in addition to standard of care, on cardiometabolic outcomes in 4017 patients with AMI and increased risk for subsequent development of HF without diabetes or chronic HF.^64^ After 1 year, the analysis of the primary hierarchical composite outcome resulted in significantly more wins for dapagliflozin than for placebo (win ratio, 1.34; 1.20–1.50; *P* < 0.001), mainly driven by the added cardiometabolic outcomes. However, dapagliflozin did not impact the composite of CV death or HF hospitalization (0.95; 0.64–1.40) and other CV events.

In patients with AMI, the severity of left ventricular (LV) dysfunction and the presence of congestion are associated with higher mortality and more frequent readmissions, especially for HF.^65^ The EMPACT-MI trial found that in 3260 patients with AMI with LV dysfunction (78% with LVEF <45%) or signs or symptoms of congestion (40% on loop diuretics, and ∼50% with NT-proBNP levels ≥1400 pg/mL), early initiation (within 14 days of admission) of empagliflozin (10 mg o.d.) did not reduce the risk of the composite primary endpoint (HF hospitalization and all-cause mortality) compared with placebo during a median follow-up of 17.9 months (HR 0.90; 0.76–1.06; *P* = 0.21).^66^ These results were consistent across the spectrum of LVEF and congestion risk profiles. In a pre-specified secondary analyses of this trial, treatment groups were analysed for HF outcomes.^67^ Over a median follow-up of 17.9 months, as compared with placebo, empagliflozin significantly reduced the risk of first (HR 0.77; 0.60–0.9; *P* = 0.03) and total HF hospitalizations (0.67; 0.51–0.89; *P* = 0.006). Empagliflozin also reduced the risks of first HF hospitalization or death due to HF (0.78; 0.62–0.98; *P* = 0.031) and total HF hospitalizations or death due to HF (0.69; 0.51–0.93; *P* = 0.015). This benefit was consistent in patients with STEMI or non-STEMI (NSTEMI) and with or without T2D. Among patients discharged while not being on diuretic therapy, significantly fewer patients in the empagliflozin group were started on a diuretic other than MRAs within 6 months post-discharge as compared with placebo; similarly, fewer patients were initiated on renin–angiotensin inhibitors, sacubitril–valsartan, or MRAs among patients not on these therapies at discharge (all *P* < 0.05). These results suggest that although empagliflozin did not reduce the risk of death after MI, it may have a potential role in preventing HF hospitalizations.

### Colchicine

The CHANCE-3 trial showed that in patients with minor-to-moderate IS or TIA and high-sensitivity C-reactive protein levels ≥2 mg/L, early initiation of colchicine (0.5 mg b.i.d. on days 1–3, followed by 0.5 mg o.d. thereafter) was not superior to placebo in reducing the risk of subsequent stroke within 90 days (HR 0.98; 0.83–1.16; *P* = 0.79).^68^ However, the short follow-up and the lower limit 95% CI of the HR suggest that sustained treatment may be required for secondary prevention. This possibility was studied in the CONVINCE trial, which investigated whether long-term colchicine (0.5 mg/day for 33.6 months) added to guideline-based usual care would reduce recurrent vascular events in 3144 patients with non-severe IS or high-risk TIA.^69^ Patients on colchicine had numerically fewer recurrent stroke and coronary events compared with those on guideline-based therapy only, but differences were not statistically significant for the intention-to-treat analysis. However, COVID-19 pandemic and budget limitations resulted in the termination of the trial before the originally planned full cohort follow-up was completed. Thus, the role of colchicine in this population remains uncertain.

The CLEAR SYNERGY trial, the largest colchicine trial so far enrolling 7062 patients, evaluated the potential benefit of colchicine on CV outcomes in patients with MI (NSTEMI 5% and STEMI 95%) who had undergone PCI.^70^ After a median follow-up of 3 years, colchicine (0.5 mg b.i.d. for the first 3 months for patients weighing ≥70 kg, followed by o.d. dose) vs. placebo did not reduce the composite of CV death, recurrent MI, stroke, or unplanned ischaemia-driven coronary revascularization and the incidence of individual components was similar in the two groups, but reduced the C-reactive protein levels at 3 months (least-squares mean difference −1.28 mg/L). Diarrhoea was more frequent with colchicine than with placebo (*P* < 0.001), but the incidence of serious infections did not differ between groups.

### Renin–angiotensin–aldosterone system inhibitors

The PARADISE-MI trial compared sacubitril/valsartan and ramipril in 5661 patients stratified according to AMI type (76% with STEMI and 24% with NSTEMI) complicated by LV dysfunction and/or pulmonary congestion and at least one risk-enhancing factor.^71^ Compared with ramipril, sacubitril/valsartan did not reduce the primary endpoint of CV death or incident HF in patients with STEMI (HR 0.87; 0.73–1.04; *P* = 0.13) and NSTEMI (HR 0.97; 0.75–1.25). Thus, sacubitril/valsartan was not superior to ramipril in this population, irrespective of the type of AMI.

Many patients undergoing major surgery are treated with angiotensin-converting enzyme inhibitors (ACEIs) or angiotensin receptor blockers (ARBs), which might lead to intra-operative hypotension, post-operative CV events, and acute kidney disease. The STOP-or-NOT trial randomized 2222 patients receiving ACEIs (46%) or ARBs (54%) for at least 3 months to continue or to discontinue their use 48 h before major non-cardiac surgery.^72^ Rates of all-cause mortality and major post-operative complications were similar in the ACEI/ARB discontinuation and continuation groups. Episodes of hypotension during surgery were more frequent (41% vs. 54%) and longer in the ACEI/ARB continuation than in the discontinuation group, but a continuation strategy was not associated with a higher rate of post-operative complications. Although both strategies are acceptable and safe, a continuation strategy may be preferred for practical purposes.

The CLEAR SYNERGY trial evaluated the potential benefit of spironolactone on CV outcomes in patients with MI (STEMI/NSTEMI 95%/5%) who had undergone PCI. At 3 years, spironolactone, as compared with placebo, did not reduce the co-primary outcome 1, composite of CV death or new or worsening HF (aHR 0.91; 0.69–1.21; *P* = 0.51), or the co-primary outcome 2, composite of first occurrence of MI, stroke, new or worsening HF, or CV death (0.96; 0.81–1.13; *P* = 0.60).^73^ Hyperkalaemia leading to drug discontinuation and gynaecomastia were significantly more frequent in the spironolactone group. Thus, in patients with MI spironolactone did not reduce the incidence of a broad composite of CV outcomes.

### Diuretics

The primary goals during acute decompensated HF (ADHF) hospitalization are decongestion with loop diuretics and guideline-directed medical therapy (GDMT) optimization. The DICTATE-AHF trial compared the efficacy and safety of adding dapagliflozin IV loop diuretics initiated within 24 h in patients with T2D hospitalized with hypervolaemic ADHF and an eGFR >30 mL/min/1.73 m^2^ until day 5 or hospital discharge.^74^ There was no difference in diuretic efficiency (primary endpoint) between dapagliflozin (10 mg/day) and usual care, but dapagliflozin was associated with reduced loop diuretic doses (560 mg vs. 800 mg; *P* = 0.006) and fewer i.v. diuretic up-titrations (*P* ≤ 0.05) to achieve equivalent weight loss as usual care. Dapagliflozin also improved median 24-h natriuresis (*P* = 0.03) and urine output (*P* = 0.005), expediting hospital discharge over the study period. Thus, although the primary outcome did not achieve statistical significance, early initiation of dapagliflozin during ADHF hospitalization is safe and fulfils a component of GDMT optimization.

### Icosapent ethyl

The RESPECT-EPA trial assessed the clinical benefits of icosapent ethyl (IPE, 1800 mg daily) in 2506 Asian patients (17% females) with CAD and low eicosapentaenoic acid (EPA)/arachidonic acid ratio (mean 0.243) treated with statins for at least 1 month.^75^ Over a median period of 5 years, IPE resulted in a numerically lower risk of CV events (CV death, non-fatal MI, non-fatal IS, unstable angina, and coronary revascularization) that did not reach statistical significance (HR 0.79; 0.62–1.00; *P* = 0.055). However, the secondary composite endpoint of coronary events (sudden cardiac death, fatal and non-fatal MI, unstable angina requiring emergency hospitalization, and coronary revascularization) was significantly lower in the EPA group (0.73; CI 0.55–0.97). Gastrointestinal disorders (*P* < 0.001) and new-onset AF (3.1% vs. 1.6%; *P* = 0.017) were higher in the EPA group. These results are consistent with previous trials showing a beneficial effect of IPE on CV endpoints as shown in a large meta-analysis.^76^

## Drug safety studies

Several studies primarily analysed specific safety aspects of several drugs clinically relevant to optimize the treatment in patients with CVD (*Table 3*).

### Anticoagulant drugs

A retrospective cohort study in 204 155 Medicare beneficiaries with AF compared serious bleeding risk for new users of apixaban or rivaroxaban treated with diltiazem or metoprolol for ventricular rate control.^77^ After 1 year, patients receiving diltiazem had increased risk for the primary outcome, a composite of bleeding-related hospitalization and death with recent evidence of bleeding (HR 1.21; 1.13–1.29), and its components of bleeding-related hospitalization (1.22; 1.13–1.31) and death (1.19; 1.05–1.34) compared with patients receiving metoprolol. The risk increases at doses of diltiazem >120 mg/day (HR 1.29; 1.19–1.39) compared with lower doses (HR 1.13; 1.14–1.24). However, neither dose produced significant changes in the risk for IS/SE or death without recent evidence of bleeding. Thus, diltiazem, a CYP3A4 inhibitor, may increase blood concentration of CYP3A4 substrates such as apixaban or rivaroxaban leading to potentially relevant bleeding events. However, the limitations of this retrospective, observational, non-randomized study should be acknowledged.

A population-based cohort study using two US claims datasets compared the effectiveness and safety of apixaban vs. rivaroxaban and vs. warfarin in patients with liver cirrhosis and non-valvular AF.^78^ Rivaroxaban initiators had significantly higher rates of major haemorrhagic events (HR 1.47; 1.11–1.94) and gastrointestinal bleeding events than apixaban initiators, but there were no differences in the rates of ischaemic events or death. Warfarin initiators also had significantly higher rates of major haemorrhage than apixaban initiators (HR 1.38; 1.03–1.84), particularly haemorrhagic stroke (HR 2.85; 1.24–6.59), but no differences were observed for major ischaemic events or death between apixaban and warfarin. Thus, with the limitations of a non-randomized comparison, apixaban may be preferable over rivaroxaban or warfarin in patients with cirrhosis and AF requiring oral anticoagulation therapy. However, these results can be only applied to patients with mild/moderate liver impairment, but not to those with more severe forms of cirrhosis, where apixaban and rivaroxaban are contraindicated.

Selective serotonin reuptake inhibitors (SSRIs) are the most widely prescribed antidepressants, but increase the risk of bleeding.^79^ A population-based, nested case–control study analysed whether the use of SSRIs with OACs [both direct OACs (DOACs) and vitamin K antagonists (VKAs)] increased the risk of bleeding compared with OAC alone.^80^ Coadministration of SSRIs and OACs increased the risk of major bleeding compared with OACs alone (IRR 1.33; 1.24–1.42). The risk peaked during the first 30 days of continuous use (1.74; 1.37–2.22) and persisted for up to 6 months. The association was present both with concomitant use of SSRIs and DOACs compared with DOAC use alone (1.25; 95% CI, 1.12–1.40) and concomitant use of SSRIs and VKAs compared with VKA use alone (1.36; 95% CI, 1.25–1.47). Thus, among patients with AF, coadministration of SSRIs and OACs requires close monitoring for bleeding or bleeding prevention, particularly during the first few months of therapy.

A nationwide cohort study investigated the bleeding risk of using non-steroidal anti-inflammatory drugs (NSAIDs) in patients treated with OACs for venous thromboembolism (VTE).^81^ Compared with non-use, aHRs in NSAID users were 2.24 (1.61–3.11) for gastrointestinal bleeding, 3.22 (1.69–6.14) for intracranial bleeding, 1.57 (0.98–2.51) for urinary tract bleeding, 1.36 (0.67–2.77) for thoracic and respiratory tract bleeding, and 2.99 (1.45–6.18) for anaemia caused by bleeding. These results were consistent for anticoagulant and VTE subtypes and confirmed that the risk of bleeding was not restricted to the gastrointestinal tract but to other organ systems.

### Glucose-lowering drugs

A retrospective matched cohort study using data from a centralized data registry evaluated by neuro-ophthalmologists assessed whether semaglutide was associated with non-arteritic anterior ischaemic optic neuropathy (NAION) in patients with T2D or overweight/obesity compared with patients taking other non-GLP-1RAs.^82^ At 3 years, patients treated with semaglutide were more likely to develop NAION in patients with T2D (8.9% vs. 1.8%; HR 4.28; 1.62–11.29; *P* < 0.001) or overweight/obesity (6.7% vs. 0.8%; 7.64; 2.21–26.36; *P* < 0.001) than those using non-GLP-1 drugs. However, causality cannot be established due to the study design. Interestingly, another retrospective cohort study using data from the TriNetX Analytics Network suggested that semaglutide, compared with glucose-lowering or weight loss medications other than GLP-1RAs, did not increase the risk of NAION in patients with T2D and/or obesity over 3 years.^83^ Therefore, it may be unjustified not to administer semaglutide based on a possibly increased risk of NAION and further prospective RCTs are needed to determine the association between semaglutide use and risk of NAION.

### Other drugs

Trolle Lagerros *et al*.^84^ used the Swedish Prescribed Drug Registry to investigate the association between PDE5I treatment, nitrates, and CV outcomes in men with previous MI or PCI. Although the coadministration is contraindicated, they identified 5710 patients who received at least two prescriptions for both a PDE5I and a nitrate medication and followed them for a median of 5.9 years. Coadministration of PDE5Is with nitrates increased all-cause mortality (HR 1.39; 1.28–1.51), CV death (1.34; 1.11–1.62), non-CV mortality (1.40; 1.27–1.54), MI (HR 1.72; 1.55–1.90), HF (1.67; 1.48–1.90), cardiac revascularization (1.95; 1.78–2.13), and MACE (1.70; 1.58–1.83). However, few events occurred 28 days after dispensing the PDE5Is. The results are in line with current guidelines suggesting that in patients on chronic oral nitrate therapy, the use of PDE5Is is contraindicated.

A case–crossover study including 429 612 individuals in nationwide Danish registries analysed the associations between first-ever triptan use and ischaemic outcomes, comparing triptan exposure in the 2-week period up to the event with four 2-week reference periods.^85^ Triptan initiation was associated with higher risk of IS (OR 3.2; 1.3–8.1), ischaemic/non-specified stroke (3.0; 1.5–5.9), and MI (3.3; 1.0–10.9). Case patients had a high-risk CV profile, while in patients with low background CV risk, the risk of an ischaemic event after triptan initiation was very low. Thus, caution is advised when prescribing triptans to patients with a high-risk CV profile.

## New cardiovascular drugs on the horizon

An increasing number of drugs are currently evaluated in ongoing phase 2 (mainly dose-finding) and phase 3 trials that evaluate their efficacy and safety in the treatment of various CVDs. *Table 4* summarizes drugs and studies that in our opinion carry the greatest interest and potential for the treatment of CVD. Worth to mention: (1) the important advances in the development of new lipid-lowering and antiobesity drugs with very different mechanisms of action, including the development of dual or triagonists of the GIP, GLP-1, and/or glucagon receptors (GIPR/GLP-1R/GCGR) in the treatment of metabolic diseases and the combination of cagrilintide, a dual amylin and calcitonin receptor agonist, and semaglutide (CagriSema); (2) the progressive increase in biological medications [antisense oligonucleotides (ASOs), siRNAs, and monoclonal antibodies (mAb)] and small molecules under development. The long-lasting effect of ASOs, siRNAs, and mAb with an infrequent dosing schedule may help to address the common problem of adherence; and (3) the results of trials with coagulation factor XI (FXI) inhibitors after the early termination or negative results of some RCTs.^61,86^ Once again, the promising and sometimes exciting results of small size and dose-finding phase II trials underpowered to assess efficacy must be confirmed in larger phase 3 trials.

**Table 4:** New drugs in phase 2 and 3 clinical development

	Drug	Mechanism of action	Clinical trials (acronym, NCT Identifier Number)
Antiarrhythmics	Etripamil, nasal spray	Short-acting L-type Ca^2+^ channel blocker	Paroxysmal supraventricular tachycardia: NCT04952610; NODE-202, NCT05763953; CT05410860
	CRD-4730	CaMKII inhibitor	Catecholaminergic polymorphic ventricular tachycardia: NCT06658899, NCT06005428
Antiobesity drugs	Cagrisema	Semaglutide plus the long-acting amylin analogue cagrilintide	T2D ± obesity: NCT064037 NCT0617636561; NCT06221969; REIMAGINE 1, NCT06323174; REIMAGINE 2, NCT06065540; REIMAGINE 3, NCT06323161, REIMAGINE 5, NCT06534411; REDEFINE 2, NCT05394519. Overweight or obesity: NCT06388187; REDEFINE 1, NCT05567796; NCT05813925; NCT06207877; NCT06267092; REDEFINE 6, NCT05996848. CKD + T2D + overweight/obesity: NCT06131372
	Orforglipron (LY3502970)	GLP-1 receptor agonist	T2D: ACHIEVE-J, NCT06010004; ACHIEVE-2, NCT06192108; ACHIEVE-5, CT06109311. Obese/overweight: ATTAIN-1, NCT05869903; ATTAIN-MAINTAIN, NCT06584916. Obstructive sleep apnoea: ATTAIN-OSA, NCT06649045
	Retatrutide (LY3437943)	Agonist of GIP, GLP-1, and glucagon receptors	Obesity: TRIUMPH-1, NCT05929066; TRIUMPH-5, NCT06662383. Overweight/obesity + T2D: TRIUMPH-2, NCT05929079. Overweight/obesity + knee osteoarthritis: TRIUMPH-4, CT05931367. Obesity + CVD: TRIUMPH-3, NCT05882045; TRIUMPH-OUTCOMES, NCT06383390. Overweight/obesity + CKD ± T2D: NCT05936151. T2D: TRANSCEND-T2D-1, 2, 3 (NCT06354660, NCT06260722, NCT06297603)
	Survodutide	GLP-1 and glucagon receptor agonist	Overweight/obesity: NCT06492135; SYNCHRONIZE™JP: NCT06176365; SYNCHRONIZE™—CVOT, NCT06077864
Anticoagulants	Abelacimab (MAA868)	mAb that binds to the catalytic domain of FXI and prevents its activation	AF vs. rivaroxaban: AZALEA-TIMI 71, NCT04755283. High-risk patients with AF unsuitable for OAC: LILAC-TIMI 76, NCT05712200. Treatment of cancer-associated VTE: ASTER, NCT05171049; MAGNOLIA, NCT05171075
	ML-2060	mAb that binds and inhibits FXIa activation	ESKD on haemodialysis: NCT05027074
	REGN9933	mAb that binds and inhibits FXIa activation	Peripherally inserted central catheter: ROXI-CATH, NCT06299111
	Asundexian	Small molecule that targets the active site of FXIa and blocks its activity	AF ineligible for OAC: OCEANIC-AFINA. Secondary stroke prevention: OCEANIC-STROKE, NCT05686070
	Milvexian	Small molecule that to the active site of FXIa and blocks its activity	Post-MI: LIBREXIA-ACS, NCT05754957. Secondary stroke prevention: LIBREXIA-STROKE, NCT05702034. AF vs. apixaban: LIBREXIA-AF, NCT05757869
Antiplatelets	Glenzocimab	Humanized GPVI-specific Fab fragment 9O12 against the extracellular domains of GPVI	STEMI: LIBERATE, ISRCTN15443962
Diabetic cardiomyopathy	AT-001	Highly selective aldose reductase inhibitor	Diabetic cardiomyopathy: NCT04083339
Dyslipidaemia	Enlicitide decanoate	Oral PCSK9 inhibitor	Hypercholesterolaemia: CORALreef Lipids, NCT05952856; CORAreef-extension, NCT06492291. Versus ezetimibe ± bempedoic acid: CORALreef AddOn, NCT06450366. HeFH: CORALreef HeFH, NCT05952869. MACE: CORALreef Outcomes, NCT06008756
	Lepodisiran	siRNA targeting Lp(a)	Elevated Lp(a): NCT05565742; ACCLAIM-Lp(a), NCT06292013
	Lerodalcibep	Recombinant fusion protein of a PCSK9-binding domain (adnectin) and human serum albumin	Patients with CVD on statins: LIBerate-CVD, NCT04797247. CVD or at high-risk for CVD: LIBerate-HR, NCT04806893; LIBerate-VI, NCT05004675. HoFH, HeFH, and high-risk CVD patients: LIBerate-OLE, NCT04798430
	Muvalaplin	Oral small molecule that inhibits Lp(a) formation by blocking the apo(a)-apo B_100_ interaction	Elevated lipoprotein(a) at high risk for CV events: KRAKEN, NCT05563246
	Obicetrapib	Highly selective cholesteryl ester transfer protein inhibitor	Elevated Lp(a) levels: VINCENT, NCT06496243. Plus statins: ROSE, NCT04753606. On top of lipid-modifying therapies: BROADWAY, NCT05142722. Plus ezetimibe: TANDEM, NCT06005597. HeFH: BROOKLYN. ASCVD: PREVAIL, NCT05202509
	Olezarsen	Ligand conjugated ASO targeting apoC-III	HT: NCT05681351; CORE, NCT05079919; CORE2, NCT05552326). FCS: NCT05130450. FCS treated with volanesorsen: NCT05185843. HT and ASCVD, or severe HT: ESSSENCE, NCT05610280
	Olpasiran	siRNA that prevents assembly of Lp(a)	ASCVD: OCEAN(a) DOSE, NCT05581303
	Pelacarsen	ASO targeting the mRNA transcribed from the *LPA* gene	Elevated Lp(a) + ASCVD: Lp(a)HORIZON, NCT04023552; OLE, NCT05900141; NCT06267560; NCT05305664. Calcific aortic valve stenosis: NCT05646381
	Plozasiran (ARO-APOC3)	siRNA targeting the hepatic production of apo C-III	Mixed dyslipidaemia: NCT05413135. Severe HT: NCT06347133; SHASTA -3, NCT06347003; SHASTA-4, NCT06347016; MUIR-3, NCT06347133. FCS: PALISADE, NCT05089084; NCT05902598
	Solbinsiran	DsiRNA targeting ANGPTL3	ACS undergoing PCI: NCT06096909. Calcified aortic valve stenosis: NCT04968509
	Tafolecimab	Fully human IgG2 mAb that specifically binds to PCSK9	Before percutaneous coronary intervention in AMI: IMPROVE-AMI, NCT06683131; NCT06096909. Calcific aortic valve stenosis: NCT04968509
	Zerlasiran	siRNA to inhibit Lp(a) production	High risk for atherosclerotic CV events and elevated of Lp(a): NCT05537571
	Zodasiran	siRNA targeting *ANGPTL3* gene expression in the liver	Mixed dyslipidaemia: ARCHES-2, NCT04832971. HoFH: GATEWAY, NCT05217667
Heart failure	Mitiperstat	Selective myeloperoxidase inhibitor	HFpEF: SATELLITE, NCT03756285; ENDEAVOR, NCT04986202
	CDR132L	Specific ASO, miR-132 inhibitor	HFrEF post-MI: HF-REVERT, EudraCT number: 2021-006040-27
	Ponsegromab	Selective humanized anti-GDF15 mAb blocking GDF15/GFRAL signalling	HFrEF: GARDEN TIMI 74, NCT05492500
	Balcinrenone	MR modulator with partial antagonist activity	Chronic HF with impaired kidney function: BalanceD-HF, NCT06307652. Balcinrenone ± dapagliflozin in patients with CKD: MIRO-CKD, NCT06350123
	Ziltivekimab	Fully human mAb targeting the interleukin-6 ligand	ASCVD, CKD and inflammation: ZEUS trial, NCT05021835. CKD + Inflammation: NCT05379829. HF + inflammation: ATHENA, NCT06200207; HERMES, NCT05636176. AMI: ARTEMIS, NCT06118281
Hypertrophic cardiomyopathy	Aficamten	Cardiac myosin inhibitor	oHCM: NCT06116968; FOREST-HCM, NCT04848506; MAPLE-HCM, NCT05767346; CEDAR-HCM, NCT06412666. nHCM: ACACIA-HCM, NCT06081894
	BMS-986435/MYK-224	Cardiac myosin modulator	oHCM: NCT05667493; MERCUTIO, NCT05556343. HFpEF: AURORA-HFpEF, NCT06122779
Pulmonary hypertension	AZD3427	Relaxin agonist	HF patients and PAH Group 2: Re-PHIRE, NCT05737940
	Sotatercept	Fusion protein of the extracellular domain of the human activin receptor type IIA and the Fc domain of IgG1	PAH: MOONBEAM, NCT05587712; SOTERIA, NCT04796337; ZENITH, NCT04896008; HYPERION, NCT04811092; NCT05818137. Cpc-PH due to HFpEF: CADENCE, NCT04945460. Central cardiopulmonary performance and peripheral oxygen transport during exercise in PAH: NCT06409026
Systemic hypertension	Baxdrostat	Highly selective aldosterone synthase inhibitor	Resistant hypertension: BaxHTN, NCT06034743; BaxAsia, NCT06344104; Bax24, NCT06168409. Uncontrolled hypertension + CKD: NCT05432167
	Zilebesiran	siRNA targeting hepatic ANG mRNA expression	Mild-to-moderate hypertension: NCT06423352. High CV risk and hypertension not adequately controlled: KARDIA-3, NCT06272487
Transthyretin-mediated amyloid cardiomyopathy	Eplontersen	Ligand-conjugated ASO to inhibit the production of hepatic TTR	EPIC-ATTR, NCT06194825; CARDIO-TTRansform, NCT04136171; NCT06465810; NCT05667493
	ALXN2220	ASO that inhibits TTR	CARDIO-TTRansform, NCT06183931
	NTLA-2001	Knocking out the TTR gene	ATTR-CM, NCT06128629

ACS, acute coronary syndrome; AF, atrial fibrillation; AMI, acute myocardial infarction; ANG, angiotensinogen; *ANGPTL3*, angiopoietin-like protein-3 gene, Apo, apolipoprotein; ASCVD, atherosclerotic cardiovascular disease; ASO, antisense oligonucleotide; ATTR-CM, transthyretin-mediated amyloid cardiomyopathy; CaMKII, calcium/calmodulin-dependent protein kinase II inhibitor; CKD, chronic kidney disease; Cpc-PH, combined post-capillary and pre-capillary pulmonary hypertension; CV, cardiovascular; CVD, cardiovascular disease; DsiRNA, dicer-substrate siRNA; ESKD, end-stage kidney disease; FCS, familial chylomicronaemia syndrome; FXI, coagulation factor XI; GDF15, Growth differentiation factor 15; GFRAL, glial cell line-derived neurotrophic factor receptor alpha (GFRα)-like; GIP, glucose-dependent insulinotropic polypeptide; GLP1, glucagon-like peptide 1; GPVI, glycoprotein IV; HeFH, heterozygous familial hypercholesterolaemia; HF, heart failure; HFpEF, heart failure with preserved ejection fraction; HFrEF, heart failure with reduced ejection fraction; HoFH, homozygous familial hypercholesterolaemia; HT, hypertriglyceridaemia; IgG1/2, immunoglobulin G1/G2; L(p), lipoprotein (a); *LPA*, lipoprotein(a) gene; mAb, monoclonal antibody; MACE, major cardiovascular events; MI, myocardial infarction; MR, mineralocorticoid receptor; mRNA, messenger RNA; NCT, National Clinical Trial; nHCM, non-obstructive hypertrophic cardiomyopathy; OAC, oral anticoagulant; oHCM, obstructive hypertrophic cardiomyopathy; PAH, pulmonary hypertension; PCI, percutaneous coronary intervention; PCSK9, proprotein convertase subtilisin/kexin type 9; siRNA, small-interfering RNA; STEMI, ST-elevation myocardial infarction; T2D, type 2 diabetes; TTR, transthyretin; VTE, venous thromboembolism.

## Conclusions

We have summarized the most relevant advances in CV pharmacology in 2024 including the approval of first-in-class drugs, the label expansion for drugs already approved based on recent clinical trials, and the results of recent clinical trials with positive or negative results performed with lipid- and glucose-lowering drugs, antiobesity and antithrombotic agents, and new approaches for the treatment of patients with HF (GLP-1RAs, finerenone, SGLT2Is, or tirzepatide). The adverse drug reactions and putative drug–drug interactions of some CV drugs were also discussed. Finally, we reviewed the most promising CV drugs that are investigated in current phase 2 and 3 clinical trials and that we hope may lead to the approval of new more effective and safer drugs in the prevention and treatment of CVD in the coming years.

## Supplementary Material

pvaf012_Supplemental_File

## Notes

**Conflict of interest:** G.A.: consulting and speaker fees from Amarin, CLS, Menarini, Novartis, and Novo-Nordisk. P.F.: CEO of Pharmahungary Group, a group of R&D companies. E.L.G.: speaker honoraria or consultancy fees from AstraZeneca, Bayer, Boehringer Ingelheim, Bristol-Myers Squibb, Pfizer, Novo Nordisk, Lundbeck Pharma, and Organon; investigator in clinical trials sponsored by AstraZeneca, Idorsia, or Bayer; and received unrestricted research grants from Boehringer Ingelheim. J.M.: speaker honoraria or consultancy fees from Amarin, AstraZeneca, Bayer Healthcare, Boehringer Ingelheim, Daiichi Sankyo, Janssen, and Merck Sharp & Dohme. W.P.: received research funding and consulting fees from AstraZeneca. P.S.: grants and personal fees from Boehringer Ingelheim, Bayer, and Astra Zeneca. D.D.: received honoraria from AbbVie Deutschland and Daiichi Sankyo. Others: none.

## Data availability

The data underlying this review were taken from the quoted references.
