# Correction to: New pharmacological agents and novel cardiovascular pharmacotherapy strategies in 2024

**DOI:** 10.1093/ehjcvp/pvaf046

**Published:** 2025-06-19

This is a correction to: Juan Tamargo, Stefan Agewall, Giuseppe Ambrosio, Claudio Borghi, Elisabetta Cerbai, Gheorghe A Dan, Heinz Drexel, Péter Ferdinandy, Erik Lerkevang Grove, Roland Klingenberg, Joao Morais, William Parker, Bianca Rocca, Patrick Sulzgruber, Anne Grete Semb, Samuel Sossalla, Juan Carlos Kaski, Dobromir Dobrev, New pharmacological agents and novel cardiovascular pharmacotherapy strategies in 2024, *European Heart Journal - Cardiovascular Pharmacotherapy*, Volume 11, Issue 3, May 2025, Pages 292–317, https://doi.org/10.1093/ehjcvp/pvaf012

In the originally published version of the manuscript, there were errors in two affiliations. Affiliation ^7^ should read: “Carol Davila. University of Medicine, Academy of Romanian Scientist, Bucharest, Sector 2, Romania” instead of: “Carol Davila. University of Medicine, Bucharest, Sector 2, Romania”. Affiliation ^8^ should read: “Vorarlberg Institute for Vascular Investigation & Treatment (VIVIT), 6800 Feldkirch, Austria” instead of: “Academy of Romanian Scientist, Vorarlberg Institute for Vascular Investigation & Treatment (VIVIT), 6800 Feldkirch, Austria”.

These emendations hve been made to the article.

