# Supplementary material for: New pharmacological agents and novel cardiovascular pharmacotherapy strategies in 2024
Source: Eur Heart J Cardiovasc Pharmacother. 2025 Mar 8;11(3):292–317. doi: 10.1093/ehjcvp/pvaf012 (PMC12046579; doi:10.1093/ehjcvp/pvaf012)
Supplement: pvaf012_Supplemental_File [file pvaf012_supplemental_file.docx]

## **Supplemental Table 1. Acronyms of the clinical trials**

## A DUE: Clinical Study to Compare the Efficacy and Safety of Macitentan and Tadalafil Monotherapies With the Corresponding Fixed-dose Combination Therapy in Subjects With Pulmonary Arterial Hypertension (PAH)

## ADALA: Low-Dose Direct Oral Anticoagulation vs Dual Antiplatelet Therapy After Left Atrial Appendage Occlusion

## AEGIS-II: Study to Investigate CSL112 in Subjects With Acute Coronary Syndrome

## ANNEXA-1: Trial of Andexanet Alfa in ICrH Patients Receiving an Oral FXa Inhibitor

## ARCADIA: AtRial Cardiopathy and Antithrombotic Drugs In Prevention After Cryptogenic Stroke

## ARTESIA: Apixaban for the Reduction of Thrombo-Embolism in Patients With Device-Detected Sub-Clinical Atrial Fibrillation

## ATTRibute-CM: Efficacy and Safety of AG10 in Subjects With Transthyretin Amyloid Cardiomyopathy

## AβYSS: Beta Blocker Interruption After Uncomplicated Myocardial Infarction

## BICS: Bisoprolol in COPD Study

## BPROAD: Blood Pressure Control Target in Diabetes

## CHANCE-3: Colchicine in High-risk Patients With Acute Minor-to-moderate Ischemic Stroke or Transient Ischemic Attack

## CLEAR SYNERGY: Colchicine and Spironolactone in Patients with MI /​ SYNERGY Stent Registry

## CLEAR-Outcomes: Evaluation of Major Cardiovascular Events in Participants With, or at High Risk for, Cardiovascular Disease Who Are Statin Intolerant Treated With Bempedoic Acid (ETC-1002) or Placebo

## COLCOT: Colchicine Cardiovascular Outcomes Trial

## COLOCT: Colchicine for the Stability of Coronary Plaque in Acute Coronary Syndrome

## CONVINCE: Colchicine for Prevention of Vascular Inflammation in Non-cardio Embolic Stroke

## DACAB: Different Antiplatelet Therapy Strategy After Coronary Artery Bypass Graft Surgery

## DAPA-MI: Dapagliflozin Effects on Cardiometabolic Outcomes in Patients With an Acute Heart Attack

## DEPOSITION - Decreasing Postoperative Blood Loss by Topical vs. Intravenous Tranexamic Acid in Open Cardiac Surgery

## DICTATE-AHF: Efficacy and Safety of Dapagliflozin in Acute Heart Failure

## EMPACT-MI: A Study to Test Whether Empagliflozin Can Lower the Risk of Heart Failure and Death in People Who Had a Heart Attack (Myocardial Infarction)

## EMPA-KIDNEY: The Study of Heart and Kidney Protection With Empagliflozin

## EPIC-CAD: Edoxaban Versus Edoxaban With antiPlatelet Agent In Patients With Atrial Fibrillation and Chronic Stable Coronary Artery Disease.

## ESPRIT: Effects of Intensive Systolic Blood Pressure Lowering Treatment in Reducing RIsk of Vascular evenTs (ESPRIT) Study

## FAST-MI: French Registry of Acute ST-Elevation or Non-ST-Elevation Myocardial Infarction

## FINEARTS-HF: Study to Evaluate the Efficacy (Effect on Disease) and Safety of Finerenone on Morbidity (Events Indicating Disease Worsening) & Mortality (Death Rate) in Participants With Heart Failure and Left Ventricular Ejection Fraction (Proportion of Blood Expelled Per Heart Stroke) Greater or Equal to 40%.

## FLOW: A Research Study to See How Semaglutide Works Compared to Placebo in People With Type 2 Diabetes and Chronic Kidney Disease.

## HELIOS-B: A Study to Evaluate Vutrisiran in Patients With Transthyretin Amyloidosis With Cardiomyopathy.

## MICON: Microbleeds International Collaborative Network.

## OCEANIC-AF A Study to Learn How Well the Study Treatment Asundexian Works and How Safe it is Compared to Apixaban to Prevent Stroke or Systemic Embolism in People With Irregular and Often Rapid Heartbeat (Atrial Fibrillation), and at Risk for Stroke

- OPT-BIRISK: Optimal antiPlatelet Therapy for High Bleeding and Ischemic RISK Patients Trial
- PARADISE-MI: Prospective ARNI vs ACE Inhibitor Trial to DetermIne Superiority in Reducing Heart Failure Events After MI
- PRECISION: A Research Study to Show the Effect of Aprocitentan in the Treatment of Difficult to Control (Resistant) High Blood Pressure (Hypertension) and Find Out More About Its Safety
- REDUCE-AMI: Evaluation of Decreased Usage of Betablockers After Myocardial Infarction in the SWEDEHEART Registry.
- RESPECT-EPA: Randomized trial for Evaluation in Secondary Prevention Efficacy of Combination Therapy - Statin and Eicosapentaenoic Acid
- SELECT: Semaglutide Effects on Heart Disease and Stroke in Patients With Overweight or Obesity
- STELLAR: A Study of Sotatercept for the Treatment of Pulmonary Arterial Hypertension (MK-7962-003/​A011-11).
- STEP HFpEF DM: Research Study to Look at How Well Semaglutide Works in People Living With Heart Failure, Obesity and Type 2 Diabetes.
- STEP-HFpEF DM: Research Study to Look at How Well Semaglutide Works in People Living With Heart Failure, Obesity and Type 2 Diabetes
- STEP-HFpEF: Research Study to Investigate How Well Semaglutide Works in People Living With Heart Failure and Obesity
- STOP-or-NOT: Impact of Renin-Angiotensin System Inhibitors Continuation on Outcome After Major Surgery
- SUMMIT: A Study of Tirzepatide (LY3298176) in Participants With Heart Failure With Preserved Ejection Fraction (HFpEF) and Obesity: The SUMMIT Trial
- T-PASS (Ticagrelor Monotherapy in Patients Treated With New-Generation Drug- Eluting Stents for Acute Coronary Syndrome
- ULTIMATE-DAPT: 1-month vs 12-month DAPT for ACS Patients Who Underwent PCI Stratified by IVUS: IVUS-ACS and ULTIMATE-DAPT Trials
- VICTORION-INITIATE: A Randomized Study to Evaluate the Effect of an “Inclisiran First” Implementation Strategy Compared to Usual Care in Patients With Atherosclerotic Cardiovascular Disease and Elevated LDL-C Despite Receiving Maximally Tolerated Statin Therapy (V-INITIATE)

**Conflicts of interest**:

C. Borghi: has received lecture fees form Menarini Corporate, Servier Pharma, Amarin, Novo Nordisk, Alfasigma, Berlinchemie, Sanofi and is member of advisory board of the same companies.

E. Grove: has received speaker honoraria or consultancy fees from AstraZeneca, Bayer, Boehringer Ingelheim, Bristol-Myers Squibb, Pfizer, MSD, MundiPharma, Organon and Lundbeck Pharma. He is an investigator in clinical trials sponsored by AstraZeneca, Bayer and Idorsia, and has received unrestricted research grants from Boehringer Ingelheim.

S. Sossalla**:** has received speakers/consultancy honoraria from Boehringer Ingelheim Pharma GmbH and AstraZeneca.

JC Kaski: has received speaker honoraria from Menarini Farmaceutica s.r.l and Servier.

D Dobrev: has received honoraria for educational lectures from Novartis and Daiichi Sankyo and honoraria for consultancy services from Omeicos and AbbVie, unrelated to present manuscript.
